# Combinatorial Effects of miRNAs in HSV-2 Infection of Macrophages: An In Silico and In Vitro Integration Approach

**DOI:** 10.3390/vaccines11091488

**Published:** 2023-09-14

**Authors:** Anwesha Banerjee, Debashree Dass, Kishore Dhotre, Pooja Wakchoure, Ashwini More, Santanu Rana, Abdul A. Khan, Anupam Mukherjee

**Affiliations:** 1 Division of Virology, ICMR-National AIDS Research Institute, Pune 411026, MH, India; banerjee.anwesha1991@gmail.com (A.B.); debashree650@gmail.com (D.D.); kishoredhotre27@gmail.com (K.D.); ashwini05.s@gmail.com (A.M.); 2 Division of Microbiology, ICMR-National AIDS Research Institute, Pune 411026, MH, India; poojawakchour2203@gmail.com; 3 Department of Zoology, RPMC-University of Calcutta, Uttarpara 712258, WB, India; rana.santanu@gmail.com

**Keywords:** microRNA, HSV-2, combinatorial, inflammation, signaling, therapeutics, network biology

## Abstract

The rising issues of herpes simplex virus (HSV)-2 drug ramifications have encouraged the researchers to look for new and alternative approaches that pose minimum adversities in the host while efficiently reducing the HSV-2 infection. Although microRNAs (miRNAs), as unorthodox approaches, are gaining popularity due to eliciting highly reduced immunogenic reactions, their implications in HSV-2 research have been rarely explored. In this study, a pool of cellular miRNAs with significance in HSV-2-induced inflammatory and immune responses have been identified. Computationally recognizing the host targets of these miRNAs through network biology and machine learning, in vitro validation has been addressed along with the identification of their regulation in the HSV-2 infection. To signify the role of these identified miRNAs, they have been individually ectopically expressed in macrophages. The ectopic expression of the individual miRNAs was able to suppress HSV-2 viral gene expression. Taking a step forward, this study also highlights the Box–Behnken design-based combinatorial effect of ectopically expressed miRNAs on maximum suppression of HSV-2 infectivity. Therefore, the concentrations of each of the miRNAs optimized in a combination, predicted through expert systems biology tools were validated in vitro to not only recover the target expressions but also inhibit the HSV-2 infection in the macrophages. Overall, the study offers miRNAs as intriguing alternatives to commercially available medications against HSV-2. Moreover, the study illuminates the prophylactic potentiality of the miRNAs, which is significant since there are currently no vaccines available for HSV-2. Moving forward, the miRNAs are employed in an innovative strategy that incorporates intricate biological system models and in vitro confirmation methods to deliver a prospective combinatorial miRNA therapeutic against HSV-2 infection.

## 1. Introduction

Herpes (meaning to creep) owes its name to the spreading (creeping/crawling) nature of the skin lesions manifested after infection and is caused by the herpes simplex viruses (HSVs) in humans. HSV-2 is an enveloped DNA virus with a 152 kb double-stranded DNA genome and is the major cause of genital herpes in the worldwide population. The 74 proteins encoded by the HSV-2 genome are expressed in an orderly manner. First, the immediate early (IE) genes are expressed, which express the proteins such as the infected cell protein 0 (ICP0), necessary for the regulation of HSV-2 replication [1]. After the IE gene regulation, the early (E) genes are expressed that encode for HSV-2 proteins, such as the DNA-binding protein ICP8, participating in the DNA synthesis of the virus [2]. Finally, the late (L) genes are expressed to encode the HSV-2 structural proteins, such as glycoprotein (g)D and gB [3]. The suffering endured by individuals infected with HSV-2 and the social consequences impact the mental as well as the reproductive health of the community to which they belong. As per records published in 2016, 491.5 million people were infected with HSV-2 [4,5]. In India, the overall seroprevalence of HSV-2 infection in the general adult population ranges from 7.9% to 18.9% [6]. Thus, the World Health Organization (WHO) has raised its concerns for the increased global burden of HSV-2 infection. Currently, there is no vaccine available for HSV-2 treatment. Moreover, the problems of resistance, limitations of bioavailability, and long-term toxicity of the available drugs clearly indicate that HSV-2 research is far from reaching its culmination [7]. Therefore, the prevalence of the disease and its resistance to current remedies along with their side effects makes it imperative for researchers in this field to come up with new strategies for the prevention and management of HSV-2.

With the recent approval of the use of RNA therapeutics by the Food and Drug Administration (FDA), USA, researchers and industrialists alike are encouraging RNA therapeutics/antisense therapeutic research, because they elicit reduced side-effects and are now being chemically improved for efficient bioavailability at low concentrations. MicroRNAs (miRNAs), small, non-coding RNAs transcribed by our genome, are about 22 nucleotides in size, and have the capability to regulate the expressions of other protein-coding genes. Being natural components of our body, miRNAs are less immunogenic and therefore might serve as efficient anti-HSV-2 agents that decrease reliance on available drugs. miRNAs function in association with the Argonaute protein to assemble onto an RNA-induced silencing complex (RISC) to identify and restrict the expression of the target mRNA [8]. In the present study, we have employed tools of systems biology backed by in vitro validation studies in an attempt to identify the macrophage miRNAs and their respective targets as modulated by HSV-2 infection to reveal the potential anti-HSV-2 miRNAs that may be regulated to combat the spread of HSV-2 in the host.

## 2. Materials and Methods

### 2.1. Cell and Virus Culture

Human embryonic kidney (HEK) 293T (ATCC: CRL-3216™, ATCC, Manassas, VA, USA) and the human monocyte/macrophage type cell line THP-1 (ATCC: TIB-202™,ATCC, Manassas, VA, USA) were used for the in vitro validation and infection assays, respectively, while Vero epithelial cells (ATCC: CCL-81™) were used for the propagation of HSV-2 (ATCC: VR-734D™, ATCC, Manassas, VA, USA). Vero and HEK293T cells were cultured and maintained in DMEM supplemented with 10% fetal bovine serum (FBS) (Invitrogen, Waltham, MA, USA), 20 mM 4-(2-hydroxyethyl)-1-piperazineethanesulfonic acid (HEPES) and 1% penicillin-streptomycin antibiotic. THP-1 were cultured in RPMI 1640 supplemented with 10% FBS, 20 mM HEPES, 1 mM Sodium pyruvate and 1% penicillin-streptomycin antibiotics and differentiated to macrophages by 10 nM Phorbol 12-myristate 13-acetate (PMA) administration for 24 h followed by resting in PMA-free complete RPMI media for 24 h. HSV-2 was propagated in 2% FBS serum-starved Vero cells for 2–3 days, followed by virus titer determination using plaque assay as per the established protocol [9].

### 2.2. miRNA Microarray

THP-1 macrophages infected with HSV-2 for 8 h were investigated against mock-infected cell lysates for the dysregulation of cellular miRNAs involved in inflammation. Mock infection was performed by exposing cells to RPMI 1640 medium with 2% FBS. The same procedure for mock infections was followed for all the assays. Custom-made microarray polymerase chain reaction (PCR) plates specific for human inflammatory pathway (Qiagen, Hilden, Germany: MIHS-105ZA) were used for the assay. A total RNA of 1 μg was extracted by organic extraction using the TRIzol reagent as per the manufacturer’s protocol (Invitrogen, Waltham, MA, USA: 15596026). cDNA was synthesized using the miScript II RT kit (Qiagen, Hilden, Germany: 218160). The synthesized cDNA was further diluted and used as a template for the SYBR green-based quantitative (q)PCR using the miScript SYBR Green PCR kit (Qiagen, Hilden, Germany: 218073). qPCR reactions were run at 95 °C for 15 min followed by 94 °C for 15 s, 55 °C for 30 s and 70 °C for 34 s for 40 cycles. The data acquired was analyzed using the GeneGLobe data analysis tool from Qiagen (https://geneglobe.qiagen.com/in accessed on 12 August 2020).

### 2.3. TaqMan-Based Quantitative PCR for miRNA Expression Validation

The miRNAs identified via miRNA microarray that showed higher statistical significance were further validated and characterized individually in HSV-2 infected THP-1 cells by TaqMan chemistry based real-time PCR. Small RNAs including our miRNAs of interest were isolated using the mirVana™ miRNA Isolation Kit (Ambion, Austin, TX, USA: AM1560) as per the manufacturer’s protocol. Commercially available specific primer–probe TaqMan assays: hsa-miR-374a-5p (Assay id: 000563), hsa-miR-195-5p (Assay id: 000494), hsa-miR-181a-5p (Assay id: 000480), hsa-miR-29b-3p (Assay id: 002165), and hsa-miR-211-5p (Assay id: 000514) were used for individual miRNAs for their validation. RNA was further diluted to the final concentration of 2 ng for reverse transcription and real-time PCR. RNA-U6 was used for normalization of miRNA expression. The fold change in gene expression was calculated using the formula, 2^−ΔΔCt^ and the values were plotted on a graph.

### 2.4. SYBR Green-Based Quantitative PCR for Viral Gene Expression

Total RNA isolated using the TRIzol organic extraction method was used to prepare cDNA using the SuperScript™ III First-Strand Synthesis System (Invitrogen: 18080-051) with random hexamers. cDNA from total RNA extracted from the HSV-2 infected THP-1 cells was used as a template to amplify the viral gene UL30 for confirmation of HSV-2 infection using the 2X GoTaq^®^ qPCR SYBR Green Master mix (Promega, Madison, WI, USA: A6001) and 200 nM primer specific to the gene to be amplified. Also, the reduction in the viral mRNAs UL30, RL2, and UL44 was checked post-miRNA mimic/inhibitor transfection to determine the reduction in HSV-2 infection in macrophages. Reactions were run under the conditions: 95 °C for 2 min followed by 40 cycles of 95 °C for 15 s and 60 °C for 60 s. All qPCR assays were performed according to the MIQE guidelines. GAPDH was used as an internal control for normalization of the viral genes. The relative fold change in the mRNA levels was calculated using the formula, 2^−ΔΔCt^ and the values were plotted on a graph, expressing the fold changes relative to that of the controls in the respective assays. All the percentage inhibition and viral load of HSV-2 are represented in comparison to the respective controls in the assay only and are not based on the absolute quantification of the mRNA transcripts. The qPCR primers used for the amplification of the HSV-2 viral genes are listed in Supplementary Table S1.

### 2.5. miRNA Target Prediction

The selected miRNA targets were screened from miRDB, which is an online database of miRNA target prediction (https://mirdb.org accessed on 17 August 2021) [10]. The 6 targets of the 4 upregulated miRNAs and 1 downregulated miRNA that were validated in vitro were predicted and screened from this database. All upregulated miRNA targets were analyzed for common interactions with all or multiple miRNA.

### 2.6. Construction of miRNA Target Interaction Network

The target interaction network was prepared using STRING (https://string-db.org/ accessed on 17 August 2021) and visualized through Cytoscape v 3.8.0 (https://cytoscape.org/release_notes_3_8_0.html accessed on 17 August 2021) [11,12]. The confidence cutoff score 0.40 was used to filter interaction on the basis of STRING interaction database [11]. Cytoscape plugin STRING was used to filter nodes and their different network biological parameters were computed in order to analyze their role in target interaction network.

### 2.7. Screening of Common Interacting Targets and Network Biological Parameters

The redundant targets of the overexpressed miRNAs were removed and miRNA targets commonly interacting with all or at least 3 overexpressed miRNAs and 1 repressed miRNA were separated from the whole target data. The node degree value of miRNA targets in the interaction network was calculated to identify hub proteins. The degree value is an important parameter in network biology and calculates the number of incoming edges a target node has. Therefore, it indicates the centrality of a particular node in an interaction network.

### 2.8. Functional Annotation of miRNA Targets

Functional annotation of miRNA targets was performed using the DAVID Functional Annotation Tool (DAVID Bioinformatics Resources 6.8, NIAID/NIH at https://david.ncifcrf.gov/ accessed on 20 September 2021) [13]. Separate analyses for targets of the over- and under-expressed miRNAs were performed and the representative results were compared against different databases. The annotation against BBID (http://bbid.grc.nia.nih.gov accessed on 20 September 2021), BIOCARTA (http://www.biocarta.com/ accessed on 20 September 2021), KEGG (https://www.genome.jp/kegg/pathway.html accessed on 20 September 2021) and REACTOME (https://reactome.org/ accessed on 20 September 2021) pathway databases were further separated from the whole annotation chart to find the important pathways associated with the targets sets of the over-expressed miRNAs and the repressed miRNAs.

### 2.9. Cloning and Transfection

The 3′ UTR target sequences of the genes PIK3R1, AKT1, IL10, CASP3, SOS1 and CAV1, which were predicted to be the targets of miR-195, miR-181a, miR-374a, miR-29b and miR-211 were PCR-amplified with the help of cloning primers (Supplementary Table S2) which contained the restriction sites of the restriction enzymes Mlu1 (New England Biolabs, Rowley, MA, USA: R0198S) and HindIII (New England Biolabs, Rowley, MA, USA: R0104S). The PCR products were then digested with the same enzymes. The pMIR-REPORT miRNA expression luciferase reporter plasmid vector (Ambion, Austin, TX, USA: AM5795) was also digested with the same vector. Further, the digested 3′ UTR sequences were ligated into the Mlu1/HindIII site of the pMIR-REPORT plasmid vector using the T4 DNA Ligase (New England Biolabs, Rowley, MA, USA: M0202S0) at 16 °C, overnight. The ligated products were transformed into the bacterial DH5α competent cells and allowed to grow for 1 h at 37 °C in a shaker incubator. Bacterial agar plates were prepared for the isolation of single colonies of transformed bacterial cells. These colonies were further propagated in broth culture followed by plasmid isolation using the QIAprep Spin Miniprep Kit (Qiagen, Hilden, Germany: 27106). These plasmids containing the cloned 3′ UTR of the target genes were transfected or co-transfected with the mimics or inhibitors of miR-211, -195, -181a, -374a, -29b or scrambled miRNA at a concentration of 5–50 nM in the HEK293T or THP-1 cells, with the help of Lipofectamine 2000 (Invitrogen, Waltham, MA, USA: 11668019), according to the instructions from the manufacturer. The miRNA mimics and inhibitors were purchased from Thermo Fisher Scientific, Waltham, MA, USA, the catalog numbers of which are: hsa-miR-195-5p mimic (MC10827), inhibitor (MH10827), hsa-miR-181a-5p mimic (MC10421), inhibitor (MH10421), has-miR-374a-5p mimic (MC10112), inhibitor (MH10112), hsa-miR-29b-3p mimic (MC10103), inhibitor (MH10103), hsa-miR-211-5p mimic (MC10168), inhibitor (MH10168). The mimics are chemically modified, stable, double-stranded RNA molecules designed to mimic the endogenous miRNAs, whereas the inhibitors are single-stranded.

### 2.10. Luciferase Reporter Assay

For the validation of the targets of the miRNAs, the cloned 3′ UTRs of the respective target genes were cloned into the pMIR-REPORT plasmid vector and transfected or co-transfected with scrambled miR or the respective miRNA mimics of miR-211, miR-195, miR-181a, miR-374a or miR-29b into the HEK293T cells. The expression of the target genes was measured in terms of the relative luciferase activities in the target only transfected as well as the target and miRNA mimic co-transfected samples using the Luciferase Reporter Gene detection kit (Sigma-Aldrich, Saint Louis, MO, USA: LUC1-1KIT) as per the manufacturer’s instructions. A decrease in the relative luciferase activity due to the suppression of target expression by the co-transfected miRNA mimic confirms that the miRNA targets the 3′ UTR of the specific target.

### 2.11. Immunoblotting

After the experimental endpoints were achieved, the cells were washed with ice cold phosphate buffered saline (PBS) and were then lysed with RIPA buffer. These lysed samples were then run on a 10% polyacrylamide gel for the separation of proteins through electrophoresis. The separated proteins were transferred onto the PVDF membrane and the membrane was immersed in a 5% blocking solution or non-fat dried milk (NFDM) for 1 h at room temperature. The blocked membrane was then probed with monoclonal antibodies specific to the proteins PI3K p85 (Cell Signaling Technology, Danvers, MA, USA: 4257), AKT1 (Cell Signaling Technology, Danvers, MA, USA: 2938), IL10 (Santa Cruz Biotechnology, Santa Cruz, CA, USA: 8438), JAK1 (Cell Signaling Technology, Danvers, MA, USA: 3344), Caspase 3 (Cell Signaling Technology, Danvers, MA, USA: 9662), SOS1 (Cell Signaling Technology, Danvers, MA, USA: 5890), CAV1 (Cell Signaling Technology, Danvers, MA, USA: 3267) and HSV-2 ICP8 (Santa Cruz Biotechnology, Santa Cruz, CA, USA: 56992). The proteins bound to the primary antibodies, which in turn were either themselves tagged with horseradish peroxidase (HRP), or bound to secondary antibodies tagged with HRP, were observed after the addition of HRP substrate Pierce™ ECL Western Blotting Substrate (Thermo Scientific, Waltham, MA, USA: 32209) in the Chemidoc imaging system (BioRad, Hercules, CA, USA). All PVDF membranes were stripped using 1× ReBlot Plus Strong Antibody Stripping Solution (Merck-Millipore, Burlington, MA, USA: 2504), blocked and re-probed with primary antibody specific to the internal control GAPDH (Santa Cruz Biotechnology, Santa Cruz, CA, USA: 47724-HRP). The protein band intensities were measured using the ImageJ software v1.53a (NIH) and were normalized with GAPDH. The values displayed in the immunoblots are representative of the relative fold changes in the signal generated, implicating meaningful alterations in the protein expressions relative to their respective controls, which have been denoted as 1.00. All the immunoblots presented in the manuscript were compliant with the integrity policies.

### 2.12. Plaque Reduction Assay

The miRNA combination, scrambled miRNA and transfection control, or untransfected monolayers of Vero epithelial cells were infected after 48 h post-transfection, with known serial dilutions of HSV-2 virus stock (1 MOI). After 1 h of adsorption, the cell monolayers were overlayed with 1% Agarose in DMEM, and allowed to be incubated for 48 h. After incubation, the monolayers were stained with 0.2% crystal violet solution such that the pfu/mL was determined, clearly indicating the reduction in the number of infectious virus particles, in terms of pfu/mL, upon pre-treatment with the optimized miRNA combination.

### 2.13. Box–Behnken Design (BBD) Based Design of Experimental Model

An initial in vitro HSV-2 inhibition assay was performed and the results were used to perform the Box–Behnken design (BBD) analysis. miRNA mimic/inhibitor concentrations of as low as 5 nM and as high as 50 nM were transfected into THP-1 macrophage cell line and allowed to incubate for 48 h. Following transfection, these cells were infected with 1 MOI of HSV-2 for 8 h after which RNA was extracted for cDNA synthesis and subsequent SYBR green-based qPCR. The optimization study was performed using BBD based on a 5-factor design [14]. This study was performed using Designer Expert version 13 (https://www.statease.com/software/design-expert/ accessed on 25 January 2023). The design included a total of 43 runs including 6 center points. For the analysis lower and higher concentrations were selected and the details of design parameters are represented (Supplementary Table S3). The response values of the 43 runs were taken from experimental data. Based on the model fit analysis, the linear model was selected for further analysis.

### 2.14. Optimization and Validation of the Applied Model

ANOVA was used for the statistical validation of the model created by Design-Expert^®^ (Minneapolis, MN, USA). All the responses were fitted to linear models then evaluated in terms of the statistical significance of coefficients and R^2^ squared values. The software obtained three-dimensional response surface plots. A total of 5 optimized concentrations were selected for the validation of the chosen experiment. The optimized concentrations were formulated and characterized for the response. The perturbation graph showing most the optimal inhibition percentage was obtained. The optimum concentration at which the highest inhibition percentage was obtained was further analyzed for in vitro investigation.

### 2.15. Cell Viability Assay

The MTT (3-[4,5-dimethylthiazol-2-yl]-2,5 diphenyl tetrazolium bromide) assay (Sigma-Aldrich, St. Louis, MO, USA) was used to assess the cytotoxicity of the optimized miRNA combination in the THP-1 and Vero cells. Concentrations within the range of 12.5–800 nM were transfected to 1 × 10^4^ differentiated THP-1 as well as Vero cells in each well in 96-well plates and incubated for 48 h at 37 °C in 5% CO_2_. Untransfected, mock-transfected or scrambled miR-transfected cells were used as controls for the assay. Cell viability was evaluated after addition of 20 μL (5 mg/mL) MTT reagent. A multimode plate reader was used to measure the optical density (OD) at 550 and 630 nm. The cell cytotoxicity or CC_50_ values were determined by determining the concentration at which 50% of the cells remain viable post-transfection of the miRNA combination [15,16].

### 2.16. Statistical Analyses of In Vitro Analyses

Mean and standard deviations of a minimum of two independent experiments were represented as the final results of the study in the manuscript as mean ± standard deviation (SD). A Student’s *t*-test was performed to evaluate the differences between the two means. The *p*-value is represented in asterisks and was observed to be <0.05 in all the experiments conducted for this manuscript. The number of asterisks is indicative of the significance level, i.e., * *p* < 0.05, ** *p* < 0.01, *** *p* < 0.001. The post-test corrections for multiple comparisons following the ‘Bonferroni posttests’ and ‘Newman-Keuls Multiple Comparison Test’ involving the mock-infected and the infected experimental groups was conducted using GraphPad Prism version 5.01 (https://www.graphpad.com accessed on 13 August 2023).

## 3. Results

### 3.1. miRNAs of the Human Inflammatory Response Pathway Are Differentially Expressed in the HSV-2 Infected Macrophages

**Figure 1:**
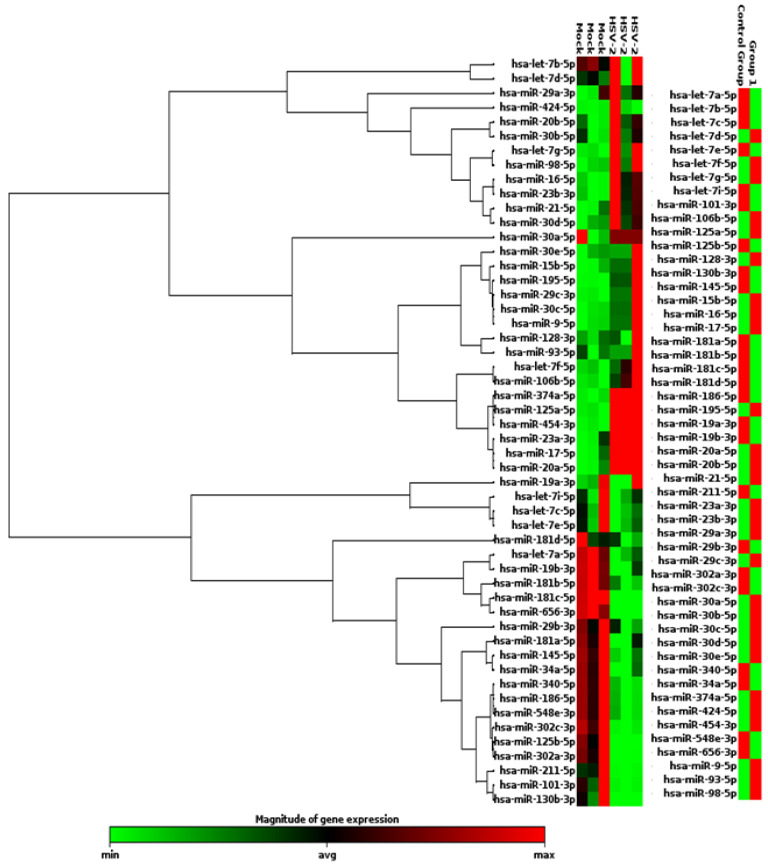
Heat map analysis of the 43 dysregulated miRNAs in the human inflammatory response pathway-focused miRNA microarray assay using control vs. HSV-2 infected samples. In the heat map, an induction in miRNA expression is indicated in red, whereas reduction in miRNA expression is indicated by green, where the change in color intensity corresponds to the expression extremities. A total of 43 miRNAs were observed to be differentially expressed between the control and the HSV-2 infected samples.

According to the miRNA database miRBase v. 22 (https://www.mirbase.org/ accessed on 15 November 2022), around 2600 miRNAs are encoded by the human genome, which makes it practically unfeasible to screen all the miRNAs in our infection model. Since it has been well established that reducing the HSV-2-induced inflammation in the female genital tract can curb the severity of HSV-2 infection, we focused our study on the miRNAs involved in the inflammatory pathway. Although the exact participation of inflammation in the HSV-2 infection has yet not been elucidated, studies have associated HSV-2-triggered inflammation with the increase in the area of the infected site as well as with virus spread. Anti-HSV agents that decrease HSV infection along with inflammation have also been reported, conclusive of reduced HSV severity and improved overall health of the hosts by the minimization of host tissue damage [17,18,19,20]. Thus, the objective of our study is to identify those inflammation-associated miRNAs that are dysregulated upon HSV-2 infection, such that their ectopic expressions not only decrease the HSV-2 infection but also spare the host of excessive inflammation. Although epithelial cells are the primary target cells of the HSV-2, our study involves the investigation of a prominent immune response, i.e., inflammation, the reactions of which are set forth by the inflammatory cytokines. Since macrophages are some of the major immune cells that release these inflammatory cytokines, we conducted our studies on in vitro cultured macrophages. Therefore, a specific pathway-focused (inflammation and immunity) microarray plate was selected for the screening and identification of the miRNAs, the mock-infected macrophages as well as the macrophages infected with HSV-2 at 1 MOI (multiplicity of infection), which may be involved in the innate immune response pathways in the HSV-2 8 h infection scenario. THP-1 cells were used as our macrophage model. While it is true that these cells are less responsive to lipopolysachharide (LPS) stimulation, an important point to be considered in inflammatory studies using LPS, it has been established that PMA is the most efficient agent for obtaining monocyte-derived macrophages that are much similar to PBMC monocyte-derived macrophages [21]. Thus, in order to obtain a homogenous background reducing the variability in the infection studies, the THP-1 cell line was preferred in our study over the primary macrophages. All together 84 miRNAs were screened for dysregulation in their expressions. The microarray data, analyzed in the GeneGlobe analysis tool, revealed the dysregulation of a total of 43 miRNAs out of 84 in the HSV-2 infected THP-1 macrophages. Upon HSV-2 infection, out of the 43 dysregulated miRNAs, 23 were upregulated and 20 were downregulated in their expressions (Figure 1). For further precision, miRNAs which were dysregulated in their expressions at a minimum of 5-fold in comparison to the control/mock-infected samples were initially focused on in this study. In contemplation of the possible roles of these miRNAs, they were further classified and divided into groups based on their role in particular cellular processes or pathways (Supplementary Figure S1A). The upregulated and downregulated miRNAs in HSV-2 infected samples are denoted by different colors and represented in a heat map, scatter plot and volcano plot (Figure 1, Supplementary Figure S1). The heat map analysis shows the extent of miRNA expression represented by two distinct colors, green (indicating reduced expression) and red (indicating elevated expression), where the intensity of the colors marks the strength of miRNA expression (Figure 1). While the heat map shows distinct extremities in the expression of the miRNAs in the HSV-2 mock-infected and infected samples, the scatter plot gives a distinct difference between the magnitudes of miRNA expression in the control group and the infected group (Group 1) on a logarithmic scale (Supplementary Figure S1B). Each point in the scatter plot shows the expression of a miRNA in the Control (*X*-axis) vs. the HSV-2 infected group 1 (*Y*-axis). Similarly, the volcano plot, which is a type of scatter plot that depicts the statistical significance in terms of the *p*-value against the magnitude of the fold change, has also been represented to show the statistical significance of the data (Supplementary Figure S1C). Volcano plot analysis is only possible if there are three replicates of the data, hence it strengthens the reproducibility and precision of the results. The implication of different representations relates to the robust selection of the miRNAs for further validation which depends not only on the extent of miRNA dysregulation but also the statistical significance. The miRNAs showing maximum dysregulation in terms of fold change in expression which fall within the 5% significance level were selected for further validation using specific primer and probes to confirm their participation in HSV-2 infection.

### 3.2. miR-195, miR-374a, miR-29b and miR-181a Are Validated to Be Reciprocally Expressed to miR-211 upon HSV-2 Infection in Macrophages

**Figure 2:**
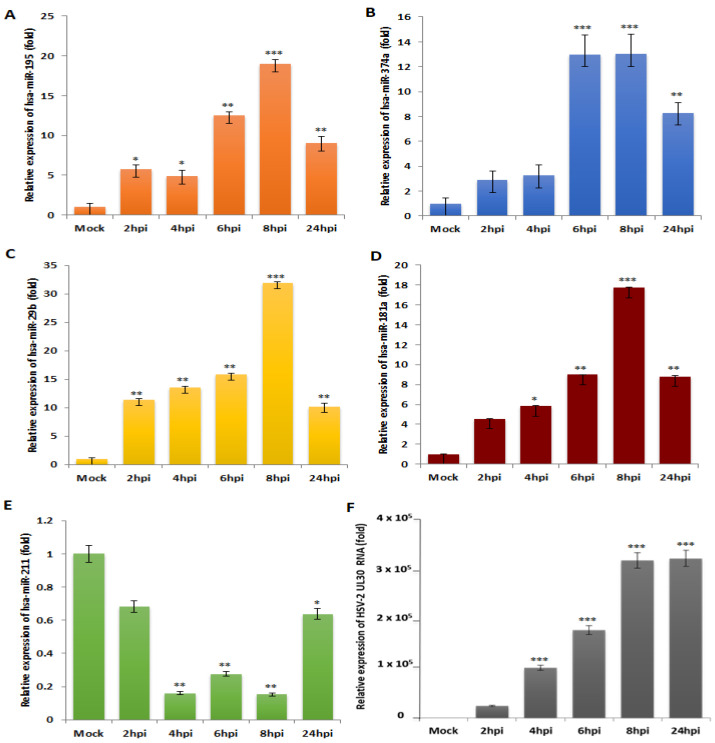
HSV-2 infection dysregulates the expressions of five miRNAs validated through qPCR. cDNA preparations of five miRNAs and the HSV-2 viral UL30 RNA were checked for their changes in expressions upon HSV-2 infection through 2–24 h using real-time PCR assays. (**A**–**D**) The expressions of the miRNAs-hsa-miR-195-5p, -miR-374a-5p, miR-29b-3p and miR-181a-5p were found to gradually increase up to 8 hpi and decrease at 24 hpi. (**E**) Expression of hsa-miR-211-5p gradually reduced up to 8 hpi but increased at 24 hpi. (**F**) The expression of the HSV-2 UL30 progressively increased up to 8 hpi and 24 hpi. The control here was mock-infected. The data represented has been statistically analyzed where the *, ** and *** represent *p* values < 0.05, <0.01 and <0.001, respectively.

Among the 43 miRNAs differentially expressed in the microarray, hsa-miR-195-5p (miR-195) was upregulated while hsa-miR-211-5p (miR-211) was downregulated with the highest fold change in expression and these were the first miRNAs selected for validation using TaqMan quantitative PCR. hsa-miR-374a-5p (miR-374a) had the highest fold upregulation, after miR-195, and was therefore also considered for validation. Along with these, miRNAs-hsa-miR-29b-3p (miR-29b) and hsa-miR-181a-5p (miR-181a) were included for validation studies to confirm their expression in the HSV-2 infected THP-1 macrophages, since these miRNAs have previously been reported to be associated with apoptosis, a cellular fate that may arise as a consequence of uncontrolled inflammation in the target cells [22,23]. Upon the screening of miRNA dysregulation, five statistically significant, differentially regulated miRNAs that demonstrated the highest fold changes in expression were validated individually using a TaqMan assay-based real-time PCR (RT-PCR or qPCR) in HSV-2 infected samples harvested at different time points of infection (Figure 2). In order to confirm the HSV-2 infection, SYBR green chemistry-based qPCR was performed for the HSV-2 UL30 viral gene, which revealed a progressive infection with time. A time kinetics of the expressions of five dysregulated miRNAs, miR-195 (Figure 2A), miR-374a (Figure 2B), miR-29b (Figure 2C), miR-181a (Figure 2D) and miR-211 (Figure 2E), along with the expression of the HSV-2 viral gene UL30 (Figure 2F) was thoroughly investigated. Interestingly, while miR-195, miR-211 and miR-374a showed similar differential expressions in the individual validation study as in the microarray, miR-29b and miR-181a were found to be upregulated in HSV-2 infected macrophages contradicting the microarray results. The four upregulated miRNAs showed a gradual increase in expression until 8 h post-infection (hpi), which decreased at 24 hpi. Similarly, the expression of miR-211 decreased until 8 hpi and restored at 24 hpi. This corresponds to the viral UL30 gene expression, which does not increase further at 24 hpi. A fold change of around10 or more in the expressions of the miRNAs upon virus infection indicates that these miRNAs might have a role to play in HSV-2 infection. Thus, the role of the miRNAs was further elucidated by the identification of the signaling cascades that these miRNAs target.

### 3.3. miRNAs Dysregulated in HSV-2 Infection Target a Vast Array of Signaling Intermediates That Participate in Several Crucial Cellular Pathways

**Figure 3:**
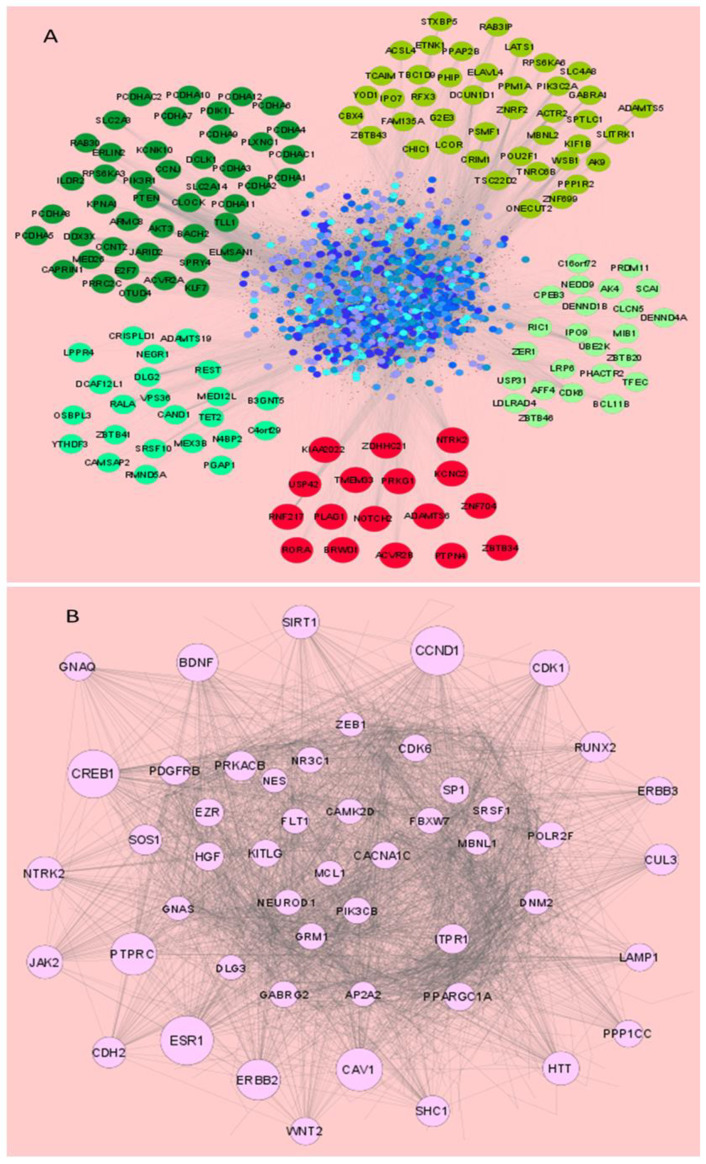
The STRING interaction networks of the miRNA targets dysregulated upon HSV-2 infection as visualized through Cytoscape v. 3.8.0. (**A**) Interaction network of the targets of the overexpressed miRNAs. While all the nodes in the figure (blue, green and red) represent the vast array of miRNA targets of the validated miRNAs, the common targets interacting with all four over-expressed miRNAs are represented by the color red, while miRNA targets interacting with any three miRNAs among the four are shown in different shades of green. (**B**) The STRING interaction network of the intermediates targeted by the under-expressed miRNA-miR-211. For ease of visualization, only the top 50 nodes with the highest degree values are displayed. The node sizes are arranged as per their comparative degree value in the interaction network.

miRNAs may have hundreds of targets in different signaling pathways. To give an idea of the extent to which miRNAs participate in the HSV-2 infection, the potential targets of these miRNAs were screened from the miRDB database. The predicted targets of the miRNAs obtained from miRDB found 1419 targets for miR-195-5p, 1340 for miR-374a-5p, 1034 for miR-29b-3p, 1408 for miR-181a-5p and 1115 targets for miR-211-5p. Therefore, a total of 5201 miRNA targets were found with the overexpressed miRNAs, and removal of the redundant targets, common among different miRNAs, found 4163 unique gene targets with the four overexpressed miRNAs. Common targets interacting with all four or at least three overexpressed miRNAs are presented in the Supplementary Table S4. A total of 17 miRNA targets were found common for all the four overexpressed miRNAs and therefore it can be assumed that these targets may be highly influenced during HSV-2 infection. An interaction network of the targets of the five dysregulated miRNAs was constructed (Figure 3). The interaction among the targets of enhanced (Figure 3A) and repressed (Figure 3B) miRNAs, prepared through the STRING database using Cytoscape v3.8, depicted the important nodes in the signaling network. The top 50 such important nodes were identified and presented here on the basis of their degree value in the interaction network analysis (Supplementary Table S5). The network analysis indicated that target nodes with high degree values also displayed high betweenness centrality levels.

The functional annotation against different pathway databases including BBID, BIOCARTA, KEGG and REACTOME using DAVID is presented as Supplementary Figure S2, through which the variety of cellular pathways hijacked by the HSV-2-manipulated miRNAs can be deduced.

Based on the results obtained from network analysis and functional annotation, six targets were selected for further interaction network analysis to elucidate their significance in our study. STRING network analysis of these targets along with other intermediates of predominant signaling cascades revealed that the selected targets are biologically significant as they are involved in the major cellular events such as inflammation, apoptosis, autophagy, etc., that govern the survival of the host cells, as well as the phagocytosis, Toll-like receptor signaling, T-cell differentiation and neutrophil extracellular trap (NET) formation, which are mechanisms of host immune cell responses (Supplementary Figure S3A). The first interacting neighbor of each of the targets was separately identified to understand that these targets can be individually or synergistically modified by their miRNA counterparts for efficient suppression of infection (Supplementary Figure S3B–G). It was also established through Cytoscape STRING analyses that AKT1 is not only the focal point of the target interaction network but also interacts with established druggable targets against different types of cancers (Supplementary Figure S4A). A visual representation of the interaction between our selected targets with other miRNAs in diseases that may become associated with HSV-2 shows that our selected targets are also involved in other sexually transmitted diseases such as human papilloma virus (HPV) and human immunodeficiency virus (HIV), which may be contracted alongside HSV-2 (Supplementary Figure S4B). Also, pathological conditions similar to HSV-2 such as cervicitis, encephalitis and inflammation involve the participation of these targets. Since these targets are involved in HSV-2-related pathology and diseases, it is possible that these targets are crucial in the pathogenesis of HSV-2. Therefore, the targets, which are integral to the inflammation and inflammation-associated pathways and have yet not been validated against our identified miRNAs in HSV-2 infection, were selected for in vitro validation and infection studies.

### 3.4. miRNAs Modulated by HSV-2 Infection Target the Components of the PI3K/AKT/mTOR Signaling Cascade

**Figure 4:**
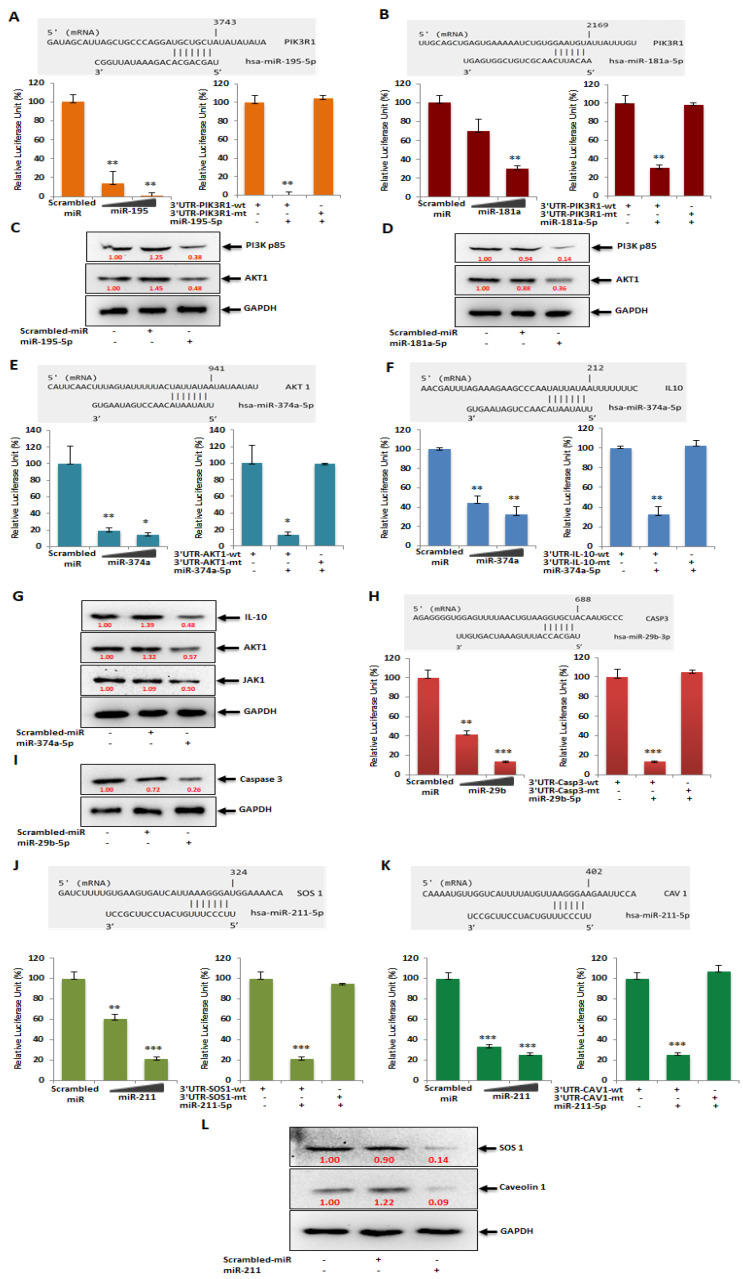
HSV-2 dysregulated miRNAs target the key intermediates in the PI3K/AKT/mTOR pathway. The 3′ UTR of the predicted miRNA target genes were cloned to confirm that (**A**–**D**) hsa-miR-195-5p and hsa-miR-181a-5p target PIK3R1, (**E**–**G**) hsa-miR-374a-5p targets AKT1 and IL10, and (**H**,**I**) hsa-miR-29b-3p targets Casp3 gene expressions. The expression of these miRNA targets is confirmed at the protein level via Western blotting after transfection with the mimics of their miRNA counterparts. hsa-miR-211-5p target SOS1 (**J**) and CAV-1 (**K**) gene expressions. The expression of these miRNA targets is confirmed at the protein level via Western blotting after transfection with the mimics of their miRNA counterparts (**L**). *, ** and *** represent *p* values < 0.05, <0.01 and <0.001, respectively.

Since miRNAs are capable of regulating a wide array of gene targets involved in various cellular events, our study emphasized the cellular genes which occupy the central position in innate immune response signaling. Phosphatidylinositol 3-kinase regulatory subunit 1 (PIK3R1), Protein kinase B (AKT1), Interleukin-10 (IL10), Caspase-3 (CASP3), Caveolin-1 (CAV1) and Son of Sevenless homolog 1 (SOS1), which are the integral signaling intermediates in the PI3K/AKT/mTOR signaling cascade, were validated as targets of the HSV-2 dysregulated miRNAs focused on in our study (Figure 4).

The minimum free energy values of the miRNA–target complex have been predicted using the miRmap web interface [https://mirmap.ezlab.org accessed on 5 February 2023] and the values indicate that these interactions may be feasible and that the complex may be stable when formed (Supplementary Table S6). The expressions of these targets were significantly suppressed when their counterpart miRNAs were transfected at a concentration of 50 nM each into the cells. A scrambled miRNA of the same concentration, having the disordered sequence of the miRNA of interest, or a mutated 3′ UTR sequence, was used as a negative control, the transfection of which could not inhibit the target expression. Reduced relative luciferase activity for each of the targets cloned into the miRNA reporter vector (pMIR-REPORT) co-transfected with their respective miRNA was revealing of the reduced target expression imposed by the specific miRNA only. The repressed protein levels of these targets further confirm the validation of the specific miRNA–target pairs. Both miR-195 and miR-181a could target PIK3R1 at different positions in the 3′ UTR with a 90% and 75% target inhibition in the luciferase reporter assay and about 62% and 86% inhibition through immunoblotting, respectively (Figure 4A–D). AKT1, which is not a target of either miR-195 or miR-181a, is suppressed upon transfection with the mimic of these miRNAs, due to the fact that it lies immediately downstream of PI3K. Thus, a decreased expression of the catalytic subunit of PI3K, i.e., PIK3R1, decreases the expression and kinase activity of the PI3K, leading to a suppressed expression of AKT1 as well, with an inhibition of 52% and 64% in the immunoblotting assays when transfected with miR-195 and miR-181a, respectively (Figure 4C,D). AKT1 and IL10 were targeted by miR-374a with a 90% and 70% inhibition in the luciferase reporter assay and about 43% inhibition through immunoblotting, respectively (Figure 4E–G). Since the interaction between IL10 and its receptor leads to the recruitment and activation of JAK1, a reduced interaction owing to the repressed expression of IL10 by miR-374a mimic, leads to a 50% reduced expression of JAK1 (Figure 4G), as well. CASP3 was validated as the target of miR-29b-3p with an 85% inhibition in the luciferase reporter assay and about 74% inhibition through immunoblotting (Figure 4H,I), whereas the downregulated miR-211 could target SOS1 and CAV1 with ~80% inhibition in the luciferase reporter assay (Figure 4J,K), and about 86% and 91% inhibition through immunoblotting, respectively (Figure 4L).

### 3.5. The miRNA Targets in the PI3K/AKT/mTOR Pathway Are Dysregulated upon HSV-2 Infection of the Macrophages

**Figure 5:**
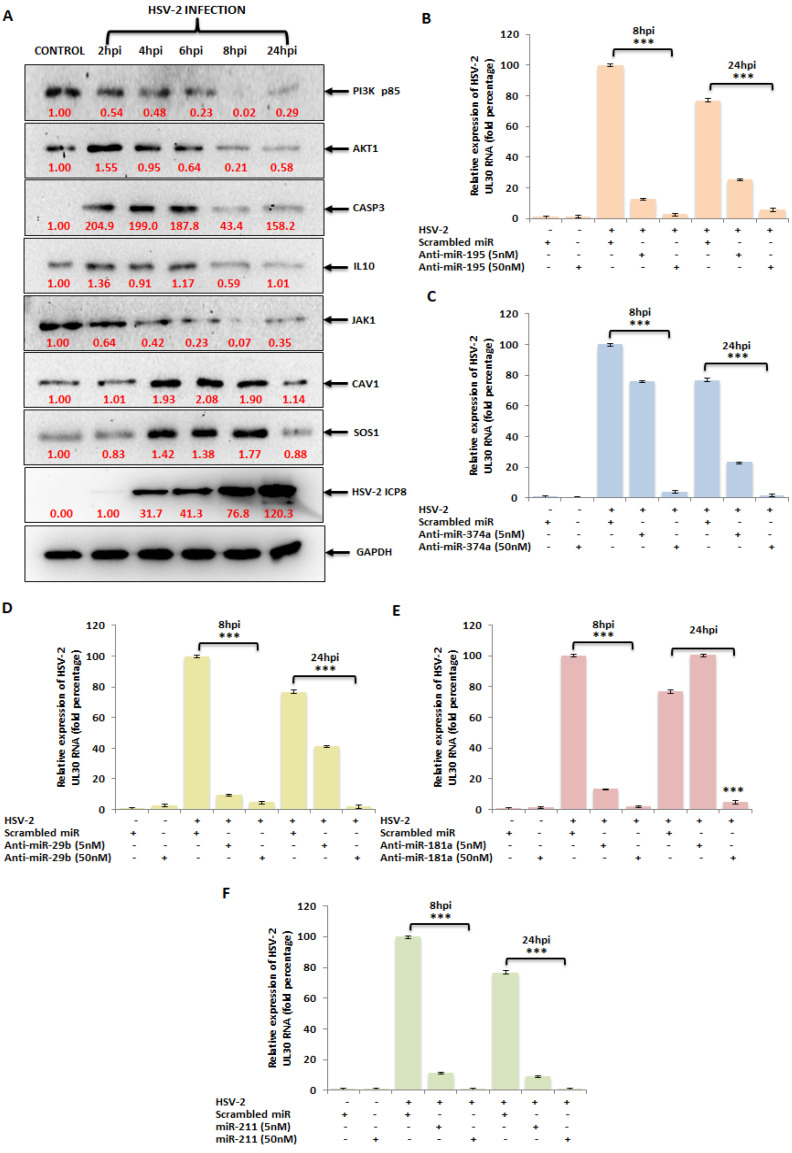
(**A**) Expression of the miRNA targets in HSV-2 infection. Protein expressions of the targets when checked upon HSV-2 infection in THP-1 macrophages using immunoblotting showed decreased target expression signals up to 8 hpi in the case of PI3K p85, AKT1, CASP3 and IL10; the miRNA counterparts of which were previously shown to be upregulated upon infection. Similarly, the expression signals of the targets of the downregulated miRNA, miR-211, were increased up to 8 hpi. Immunoblotting with HSV-2 ICP8 antibody shows a progressive infection with time. GAPDH was used as an internal control. Ectopic expressions of the validated miRNAs can inhibit the HSV-2 UL30 viral gene expression. (**B**–**E**) Shows the percentage fold change in the mRNA expression of the UL30 gene in scrambled miR or anti-miR-195, anti-miR-374a, anti-miR-29b or anti-miR-181a transfected and HSV-2 infected macrophage samples. (**F**) Represents the relative percentage fold change in the mRNA expression of the UL30 gene in miR-211 transfected and HSV-2 infected samples. The UL30 gene expression was checked against minimum 5 nM and the maximum 50 nM inhibitor/mimic concentrations. *** indicates *p* < 0.001.

In vitro target validation of the chosen miRNAs led us to investigate whether these targets are manipulated in the actual HSV-2 infection scenario. HSV-2 infection of the macrophages confirmed the deregulated expressions of these components of the PI3K/AKT/mTOR signaling cascade. With respect to the expression kinetics of the miRNAs depicted in Figure 2, the expressions of their respective targets were reciprocated (Figure 5A). Likewise, miR-195, miR-181a, miR-374a and miR-29b overexpressed during HSV-2 infection had their target expressions suppressed up to 8 hpi while the downregulated miRNA, miR-211-5p, which was getting repressed up to 8 hpi during the viral infection, had its respective target expressions upregulated up to this time-point. This signifies that the miRNA targets, which are also components of the PI3K/AKT/mTOR pathway, are manipulated by the virus itself and confirms the involvement of these components in HSV-2 pathogenesis in the macrophages.

### 3.6. Ectopic Expression of HSV-2-Dysregulated miRNAs Suppress the Expression of the HSV-2 DNA Polymerase Catalytic Subunit Gene

The in vitro target validation and infection studies prompt the most evident question of all, i.e., can the ectopic expression of the dysregulated miRNAs impact HSV-2 viral gene expression? To address this question, the HSV-2-downregulated miR-211 or the inhibitors of the upregulated miRNAs miR-195, miR-181a, miR-29b or miR-374a or scrambled miR (sc-miR) were transfected at a concentration of a minimum of 5 nM and maximum of 50 nM, followed by HSV-2 infection for 8 h or 24 h (Figure 5B–F). The respective miRNA expressions were also recorded as a measure of the transfection efficiency (Supplementary Figure S5). miRNA mimic/inhibitor transfection in the HSV-2 infected samples was capable of reducing the infection in the THP-1 macrophages. Out of the five mimic/inhibitor transfections, the anti-miR-195, -374a, -181a and the mimic miR-211 showed about a 99% inhibition in the expression of the viral DNA polymerase catalytic subunit gene (UL30) at a concentration of 50 nM at 8 hpi, when compared to the sc-miR transfected, HSV-2 infected macrophages. While the anti-miR-29b could inhibit the expression of UL30 to about 95% at the concentrations of 5 nM and 50 nM, the miR-195 and miR-181a inhibitors as well as the mimic miR-211 showed about 90% decreased UL30 expressions when transfected ectopically at a minimum of 5 nM concentration. However, inhibition by anti-miR-374a at 5 nM was observed to be as low as 20%. Meanwhile, the UL30 gene expression was observed to be inhibited 88–99% in the 50 nM inhibitor/mimic transfected HSV-2 infected macrophages at 24 hpi, 5 nM ectopic expression of each of the inhibitors could only suppress the viral UL30 gene expression to a maximum of 75%. Although 5 nM of mimic miR-211 transfections could bring down the viral gene expression of UL30 to about 94% at 24 hpi, consistent inhibitions at the 8 hpi time-point for both the 5 nM as well as the 50 nM mimic/inhibitor transfections in the infected THP-1 macrophages was recorded. A complete lytic cycle of HSV takes up to 12 h in the host cell, after which it leaves the cells causing the infected cells to undergo cell death. Most of the early replication takes place within 4–8 hpi. Moreover, by 24 hpi, HSV has already exploited the host for the establishment of pathogenesis resulting in altered regulation of many of the cellular pathways. Therefore, the attempt to restrict the HSV-2 infection during its initial replication cycle, before it weighs down on the host, resulted in selecting the 8 hpi time-point for our subsequent studies. The decrease in the gene expression of a viral mRNA that translates into the catalytic subunit of the polymerase participating in HSV-2 DNA synthesis is indicative of the modulation of HSV-2 infection by the ectopic expression of the miRNAs of interest. Having established that, we moved on in our study to observe the synergistic effect of different combinations of the mimic and inhibitors on the inhibition of HSV-2 infection in macrophages.

### 3.7. Identification of Synergistic Combination of Five Dysregulated miRNAs on HSV-2 Infection: Box–Behnken Method

The Box–Behnken design method is used for identifying optimal combinations of miRNAs concentration for maximum inhibition of HSV-2 infection [14]. A total of 43 runs were selected for the model preparation, using five-factor designs, based on the initial data observed for the suppression of HSV-2 infection obtained through in vitro analysis. The selected values for identifying the possible combinations are represented here (Supplementary Table S3). The independent variable, i.e., the R1 inhibition percentage is taken up by experimental data. Detailed information about the actual factors and their response is presented in Supplementary Table S7.

### 3.8. Dependant Variable: R1 (Response 1)—Percent Inhibition

Based on the fit summary, the linear model was selected for the present study. The details of the model fit summary are presented in Supplementary Table S8. The significant model was selected based on the sequential *p*-value and the differences between the models were adjusted and predicted based on the R^2^ value. The sequential *p*-value was observed as <0.0001, i.e., more highly significant than the other models, whereas the adjusted and predicted R^2^ value was less than 0.2, indicating that the optimal model for this study is linear.

The ANOVA (analysis of variance) test was performed on the linear model and the *p*-value of the model was determined as <0.0001, which suggests the high statistical significance of the selected model. The lack of fit of the model was identified as >0.1000, whereas the pure error was null. The model F-value of 18.08 implies the maximum significance of the model. The probability of this large F-value due noise is as minimum of 0.01%. Based on these factors, it was concluded that the model is highly significant. The detailed results of the ANOVA test are represented as Supplementary Table S9.

### 3.9. Validation of the Applied Model and Identification of Synergistic Combination of Five Dysregulated miRNAs on HSV-2 Infection

**Figure 6:**
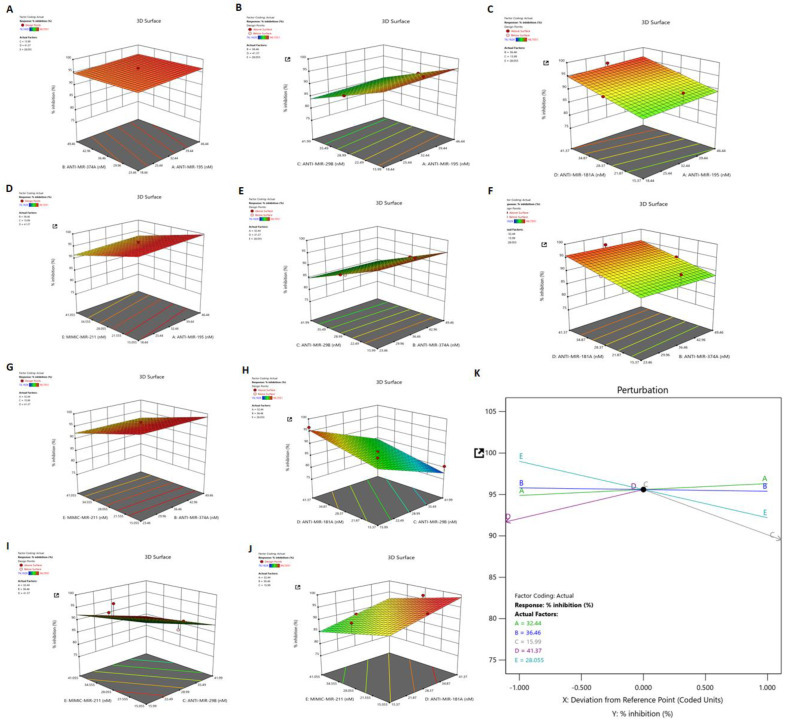
Impact of different miRNA combinations on HSV-2 inhibition. The 3D graphs display the influences of two factors on a particular response, whereas the third factor remained constant. The graphs represent the inhibition response on HSV-2 infection of the combinations (**A**) anti-miR-374a and anti-miR-195, (**B**) anti-miR-29b and anti-miR-195, (**C**) anti-miR-181a and anti-miR-195, (**D**) mimic-miR-211 and anti-miR-195, (**E**) anti-miR-29b and anti-miR-374a, (**F**) anti-miR-181a and anti-miR-374a, (**G**) mimic-miR-211 and anti-miR-374a, (**H**) anti-miR-181a and anti-miR-29b, (**I**) mimic-miR-211 and anti-miR-29b, and (**J**) mimic-miR-211 and anti-miR-181a. (**K**) The perturbation plot showing effects of different miRNAs on percentage inhibition of HSV-2. The plot also displays a point for optimum miRNA concentrations for efficient HSV-2 inhibition.

The best combinations of five miRNAs that provide maximum inhibition percentages against HSV-2 infection were identified based on the Box–Behnken design, and the combinations showing the maximum inhibition percentage were selected. The relative percentage inhibition value of the suggested 43 combinations was validated by the in vitro experimental analyses, of which 15 such combinations [C1–C15] are represented in our study (Supplementary Figure S6A,B). Using the initial in vitro validation of HSV-2 inhibition data, the effects of the combinations of five miRNAs was plotted on 3D graphs (Figure 6A–J). The best combination of factors was identified and presented in the perturbation plot generated using the Box–Behnken design method (Figure 6K). Briefly, the perturbation plot indicated an optimum factor combination giving a maximum HSV-2 inhibition percentage in the case of the present experiment. The optimized miRNA combination (C2) composed of 32.440 nM of anti-miR-195, 36.460 nM of anti-miR-374a, 15.990 nM of anti-miR-29b, 41.370 nM of anti-miR-181a and 28.055 nM of mimic miR-211 was selected amongst the other combinations (C1–C15), owing to the predicted inhibition value of 95.594%, depicting initial in vitro data of 96.705% inhibition of HSV-2 UL30 gene expression for subsequent final validation studies (Supplementary Table S10 and Supplementary Figure S6A).

### 3.10. In Vitro Combinatorial Effect of miRNAs on Attenuation of HSV-2 Infection

**Figure 7:**
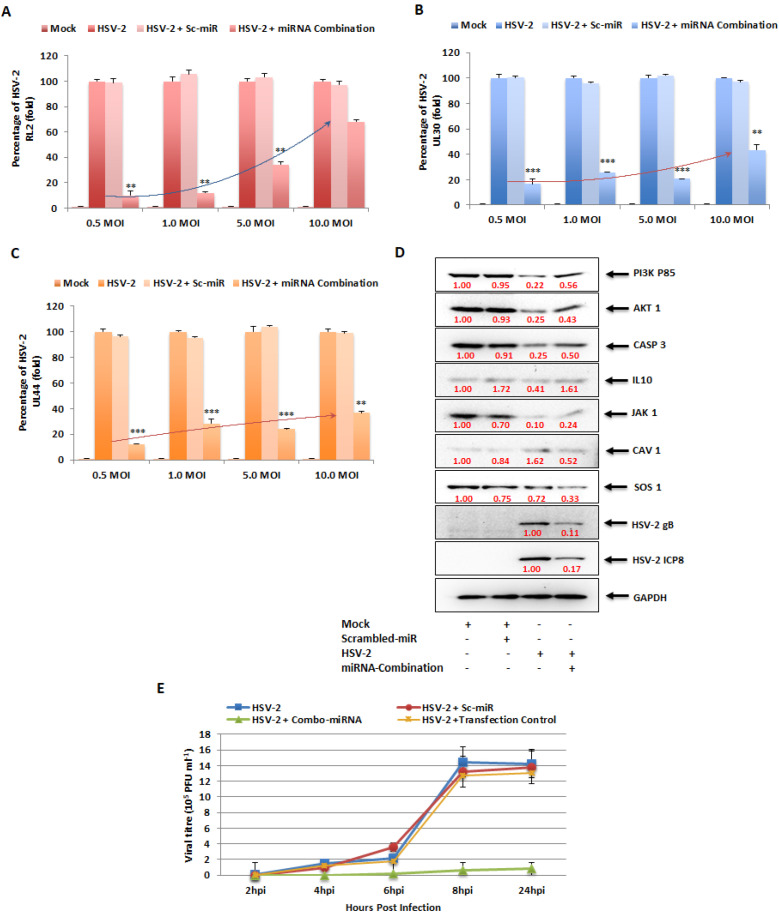
In vitro validation of the miRNA combination selected through Box–Behnken analysis. qPCR analysis of the optimized miRNA combination in the amplification of the HSV-2 (**A**) RL2, (**B**) UL30 and (**C**) UL44 genes at different MOI of 0.5, 1.0, 5.0 and 10.0. cDNA from mock-infected as well as HSV-2 infected samples transfected or untransfected with the miRNA combination were used as templates for the SYBR-green-based qPCR assays to detect the reduction in viral gene expressions. (**D**) The reduction in the viral protein products gB and ICP8 through immunoblotting. Whole cell lysates were electrophoretically separated on a 10% SDS-PAGE and transferred onto PVDF membranes to detect the protein band intensities of the respective viral proteins. GAPDH was used as the internal control in all the qPCR assays as well as the immunoblotting assay. (**E**) The virus titer of HSV-2 in presence of scrambled-miRNA or miRNA combination was examined at the indicated time points post-virus infection. Reduction in the HSV-2 titer in the presence of the miRNA combination is denoted with a green line using triplicate determinations. ** indicates *p* < 0.01 and *** indicates *p* < 0.001.

The miRNA combination optimized through the BBD was checked for cytotoxicity in the Vero and THP-1 cells (Supplementary Figure S7), and the CC_50_ value for Vero cells was found to be 176.7 nM and THP-1 cells was 169 nM. Therefore, the miRNA combination was used at 50 nM concentration, which was about 3.4-fold slower than the CC_50_ values in the two cell lines. Finally, the miRNA combination (C2) comprising the optimized concentrations of the five miRNAs of interest were validated for HSV-2 inhibition through qRT-PCR detecting the viral mRNA expression, immunoblot analysis examining the translation of viral proteins and viral load detection for identifying the number of infectious viral particle, in vitro (Figure 7). This combination contained the inhibitors of the miRNAs that were upregulated in HSV-2 infection (miR-195, miR-181a, miR-374a and miR-29b) along with the mimic of the miRNA that was downregulated in HSV-2 infection (miR-211). The combinatorial effect of these miRNA inhibitors/mimics on the expression of the HSV-2 immediate-early infection phase gene RL2, early infection phase gene UL30 and late infection phase gene UL44 was investigated. These investigations were performed at different MOIs ranging from 0.5 to 10 MOIs of HSV-2, so as to unveil the potentiality of this combination to curb HSV-2 infections presenting varied severities in the hosts. The combinatorial effect of ectopic expression of the five miRNAs of interest was observed as 84–91% inhibition in the RL2, UL30 and UL44 gene expression at 0.5 MOI, 74–88% at 1 MOI, 66–79% at 5 MOI and 32–63% at 10 MOI (Figure 7A–C). No significant effect was observed in the scrambled miR-transfected controls when compared to the mock-infected samples. The maximum inhibition was detected in 0.5 MOI, where the viral infection was comparatively lower. The protein levels of HSV-2 gB and ICP8, actively expressed during the early and late phase of infection, respectively, were considerably suppressed in the cells transfected with the selected miRNA combination. While the protein levels of gB decreased about 89%, the protein levels of ICP8 were suppressed about 83% at 8 hpi, along with around 20–50% recovery in the expressions of the miRNA targets (Figure 7D). This indicates that a moderate recovery in the expressions of the targets not only is sufficient for recovery from infection but also restricts the off-target effects that the modulation of these targets might have on the overall health of the hosts. Also, determination of the number of infectious HSV-2 viral particle, in terms of plaque-forming units per millilitre (pfu/mL), using the plaque reduction assay, was conducted to provide conclusive evidence of the substantial anti-HSV-2 effect instigated by the combinatorial therapy. Interestingly, our results indicated about a 36-fold reduction in HSV-2 viral titer (Figure 7E). A negligible effect was observed in the transfection control as well as the scrambled miR-transfected samples when compared to the HSV-2 infected, untransfected samples. Since obtaining plaques is difficult using macrophages, as macrophages do not form prominent monolayers like epithelial cells, the plaque-reduction assay was conducted in Vero cells only, whereas the reduction in infection in macrophages was investigated using real-time PCR to determine the expression of the HSV-2 viral genes. Taken together, this proves that the optimized miRNA combination decreases early HSV-2 replication and multiplication in the host macrophages.

## 4. Discussion

An insult in the form of a noxious stimuli such as HSV-2 activates the macrophages allowing it to undergo certain molecular changes leading to the release of cytokines and chemokines and, ultimately, pathogen elimination. The fact that the severity of HSV-2 could be decreased by restricting inflammation provides the basis for our emphasis on the identification of miRNAs involved in the inflammation event during HSV-2 infection [24]. As described by Orlowski et al., 2014, an ideal anti-HSV agent would be one that reduces HSV infection and regulates host inflammatory reactions at the same time. Our study identifies those miRNAs that are known to participate in inflammatory events by modulating targets involved in these inflammation-associated pathways to restrict HSV-2 infection as well as regulate inflammation in the host. This manuscript reports the potentiality of these miRNAs as anti-HSV2 agents. The role of inflammation as well as its regulation by these miRNAs in HSV-2 infection is yet to be investigated in our future studies.

While there have been numerous reports on the regulation of HSV-1 infection by host miRNAs [25,26,27,28,29,30,31,32], the same cannot be said for HSV-2. Interestingly, the host miR-138 increases HSV-1 latency by targeting the host OCT-1 and FOXC1 genes as well as the viral ICP0 gene. While the same miRNA in HSV-2 infection regulates the aforementioned genes along with the UL19 and UL20 genes, indicating that that alpha herpesviruses have evolved through HSV-1 and HSV-2 to exploit neuronal miRNAs to induce latency in the hosts [33]. It was convenient that the miRNA microarray PCR-based method could screen 84 inflammation-related miRNAs at the same time and gave us information about 43 of those miRNAs that were dysregulated upon HSV-2 infection (Figure 1). However, we focused our current study on five of the dysregulated miRNAs that have not previously been investigated in HSV-2 mediated pathogenicity. It was also observed that some of the miRNAs (miR-29b and miR-181a) showed contradictory results in the microarray and validation assays (Figure 2). It is important to note here that while the microarray is a SYBR-green-based screening method, the validation studies for these miRNAs are a more specific and cutting-edge method performed using TaqMan chemistry-based assays against the particular miRNA. Moreover, the validation assays were performed in multiple replicates such that their representation confirms their dysregulation in HSV-2 infection of the THP-1 macrophages. Due to the characteristic of miRNAs that targets more than a single gene, it is inevitable that our validated miRNAs will have numerous targets to modify. To narrow down our list of targets that need to be validated in our in vitro studies, a set of bioinformatics tools were used to predict, organize and signify the targets of these five validated miRNAs. The miRNA targets were predicted using an online database, miRDB, which identifies the common attributes associated with miRNA binding and target suppression and predicts the targets of miRNAs using machine learning methods [34].

Interaction networks constructed using the predicted miRNAs in Cytoscape provided an organized representation of the common targets (Figure 3). This information is crucial for recognizing the common pathways that may be targeted by modifying a set of miRNAs. It also helps in the identification of the ‘hubs’ in an entire interaction network, i.e., interaction network built using all the predicted targets of the validated miRNAs. The hubs are the larger nodes that have more interactions (degree) as compared to the others. With this knowledge, our search for target validation was narrowed down to those 100 nodes or miRNA target genes that had the highest degree values. At this point a functional annotation analysis proved to be useful in data compilation along with the STRING network analysis could reveal the major pathways that should be the focus of our study in terms of the target genes involved in them (Supplementary Figures S2 and S3). Therefore, our target search was further narrowed down to six targets that were directly or indirectly associated with AKT1, the node with the highest degree. Since its discovery more than 25 years ago, AKT1 has been extensively studied in many cancers as well as in other virus infections [35,36,37,38,39]. AKT and the other associated targets participate in many cellular cascades, also indicating that targeting one can sequentially affect the other. Also, involvement of these targets in neuropathological conditions suggests that these targets may have a connection to HSV-2 latency as well (Supplementary Figure S4). Therefore, the interaction networks and annotation studies in our investigation support the enormity of the cellular pathway components involved in HSV-2 pathogenecity, thus indicating the potential checkpoints that must be manipulated for anti-HSV-2 therapeutics.

Although the AKT/PI3K and mTOR pathways may be two distinct signaling networks on their own, they are intertwined in the major physiological and pathological events. The PI3K/AKT/mTOR signaling pathway plays an intricate role in macrophage activation and inflammatory effector functions, along with the vast number of cellular processes it has long been associated with [40]. Due to its involvement in macrophage purposing and therefore immunity of the host, the PI3K/AKT/mTOR signaling cascade has been the focus of our study. Hence, the miRNA targets in the PI3K/AKT/mTOR signaling cascade were validated in vitro through luciferase reporter assay and immunoblotting checking the expressions of these genes in the presence of their miRNA counterpart mimics (Figure 4). PIK3R1, an essential component of the Phosphoinositide-3-Kinase (PI3K), activates it to trigger the catalytic unit of the kinase to enable the conversion of phosphatidylinositol (3,4)-bisphosphate (PIP2) lipids to phosphatidylinositol (3,4,5)-trisphosphate (PIP3) via phosphorylation. This plasma membrane PIP3 associates with AKT to allow PDK1 to phosphorylate and activate AKT [41]. Due to this reason, the expression of the AKT1 protein was also investigated while validating PIK3R1 as a target of both miR-195 and miR-181a, as suppression of PI3K expression and activity leads to the suppression of AKT1 in the signaling cascade. AKT1, an AKT isoform predominantly present in macrophages that aids in their survival and differentiation [40,42], was the focal point of the affected signaling cascade and the direct target of miR-374a. Another target of miR-374a is IL10, the stimulation of which triggers the recruitment and activation of JAK1 tyrosine kinase, a primary component of the JAK/STAT pathway. The IL10 stimulated activation of JAK1 is also associated with the increased enzymatic activity of PI3K and AKT1 in an Adenosine-5′-monophosphate-activated protein kinase (AMPK)-dependent manner [43,44]. Consequently, the expression of JAK1 was also checked post-miR-374a transfection. AKT1, being involved in various cancers, obviously affects apoptosis [45,46,47]. Caspase 3 is a well-known effector enzyme in the apoptosis pathway and is modulated by AKT1 through the other apoptosis regulatory proteins Bax and Bcl2, which function reciprocally to regulate apoptosis [48]. This CASP3 was established as the target of miR-29b in our study. CAV1, the target of the downregulated miR-211 miRNA, is also involved in the PI3K/AKT pathway. Although there have been contradictory reports on the interaction between CAV1 and AKT, such as where CAV1 co-expresses with AKT leading to cancer progression [26], and others such as where CAV1 inhibits the AKT pathway [49]. However, the fact that CAV1 impacts the AKT pathway is undisputed. Similarly, SOS1, also a target of miR-211, impacts the expression of AKT through the activation of the proto-oncogene Ras protein [50,51]. Thus, a greater than 50% inhibition in the expressions of the targets as well as their downstream signaling proteins upon transfection of the specific miRNA mimics implies that these are strong targets of the miRNAs. If these targets actually do play a role in HSV-2 infection, the manipulation of these with their respective miRNA mimic/inhibitor may strongly regulate the viral pathogenesis for the benefit of the host. Accordingly, upon in vitro HSV-2 infection of macrophages, all targets of the upregulated miRNAs gradually decreased in their expressions with the progression of infection up to 8 hpi and were stable or slightly elevated at 24 hpi (Figure 5A). This is because the miRNAs which target these gene products substantially increased to cause extensive suppression of their targets up to 8 hpi. At 24 hpi, the miRNA expressions decreased leading to recovery of their targets. Similarly, the downregulated miRNA, miR-211-5p was decreased in its expression and was unable to suppress its targets, SOS1 and CAV1, up to 8 hpi. The suppression was observed at 24 hpi when the inhibition on miRNA itself was released. The advancement of HSV-2 infection in the THP-1 macrophages was confirmed with the expression of the HSV-2 major DNA binding, infected cell protein (ICP)-8, the expression of which was detected by 4 hpi and increased up to 24 hpi [2,3]. Although there have been some reports claiming the activation of the PI3K/AKT in HSV-2 infection, most of these studies have been conducted on epithelial cells [52,53,54]. Thus, the infected cell type and the stage of infection is also crucial in understanding the role of a signaling cascade in viral pathogenesis. Moreover, activation of the PI3K/AKT pathway has been reported to be involved in HSV-1 replication, latency and reactivation as well [55]. Despite this activation, there is report of the induction of autophagy in HSV-1 infection of THP-1 macrophages [56]. The induction of autophagy has also been implicated in HSV-2 infection through our findings. The involvement of SOS1 in HSV-2 infection has been previously reported, which supports our findings regarding this signaling intermediate [57]. Furthermore, the substantial decrease in the expression of the HSV-2 UL30 gene that encodes the catalytic subunit of the viral DNA Polymerase involved majorly in the DNA synthesis of HSV-2 is elucidative of the potentiality of these miRNAs to regulate HSV-2 infection in the host (Figure 5B–F). Having established the anti-HSV-2 potential of these miRNAs, our study proceeds towards optimization of an appropriate miRNA combination to tackle the HSV-2 infection efficiently as well as minimizing damage to the host. The same miRNA property that renders it suitable for therapeutics, also imposes a disadvantage to its application. Since miRNAs are capable of targeting hundreds to thousands of targets in the cell, the issue of off-target effects raises concerns of compromised safety in the host. A holistic approach to resolving the matter in question is the application of miRNA combinatorial therapy, where miRNAs may be synergistically employed in compositions lesser than those used in their monotherapy, to kindle an identical or improved therapeutic effect in the host. This way, the off-target effects put forth by a miRNA, otherwise beneficial, are minimized to improve the overall health of the individual. We have successfully screened a pool of such miRNA combinations to come up with an optimized miRNA combination having the highest inhibitory effect on the HSV-2 pathogenesis in the macrophages (Figure 6 and Supplementary Figure S6). The viral gene expression of RL2, UL30 and UL44 was decreased more than 95%, with a more than 80% decrease in the protein levels of the HSV-2 ICP8 and gB genes, which may be attributed to the recovery in the expressions of the miRNA targets (Figure 7A–D). Through the recovery of the targets in infection it could be inferred that the miRNA combination not only subdues the HSV-2 infection, which might be a consequence of the recuperation of the expressions of the targets, but also restricts the extreme manipulation of the signaling cascades that these targets are involved in. Thus, this provides the basis of the implementation of combination theory in our study. The RL2 gene encoding the ICP0 protein belongs to the immediate-early phase of infection, preparing the virus for the replication process. The UL30 gene encoding the catalytic subunit of the viral DNA Polymerase and the ICP8 protein encoding the DNA-binding protein participates in the replication of the HSV-2 in the cell, only after which the late infection phase proteins gC (UL44 gene) and gB are produced. Therefore, reduced gene expression of the viral proteins involved in the three phases of infection provides concrete evidence of the anti-HSV-2 potential of the selected miRNA combination. This was supported by the significantly decreased infectious HSV-2 viral particle (Figure 7E). Although it is true that the decrease in the infectious virus particle may be due to the targets of these miRNAs in the HSV-2 genome, a long line of investigation would follow such a claim as these are the predicted targets only and not the validated targets in the HSV-2 genome. Furthermore, it does not explain the decrease in the viral genes, RL2, UL30 and UL44, which are not the predicted targets (predicted using mirTar) for any of the five miRNAs used in our study. Moreover, transfection of inhibitors of the upregulated miRNAs (miR-195 and miR-181a) should only increase the expression of the predicted viral genes which would have contributed to the increase in the infectious particle count, which does not comply with the decrease observed in our results. The contribution of miR-211 utilized in combination with the other four miRNAs to exert such an effect needs to be examined, However, we do agree that employing the mimic of miR-211 could have repressed the five viral targets to contribute to the reduction in the virus titer. All-in-all, our study brings to light some of the major checkpoints that may individually or synergistically be manipulated by mechanisms of our own genome (miRNAs) to curb HSV-2 infection in the host. That being the case, it is also true that these mechanisms need to be further investigated extensively for precise targeted delivery and efficiency against HSV-2 infection in in vitro as well as in in vivo models. Therefore, contributing to the studies where other host miRNAs, such as, miR-36 inhibits HSV-2 infection by stifling the expression of the interferon induced transmembrane protein 1, or miR-138 inhibits HSV-2 infection in neuronal cells, our study provides a combination of miRNAs as a prospective anti-HSV-2 agent in macrophages [33,58].

## 5. Conclusions

**Figure 8:**
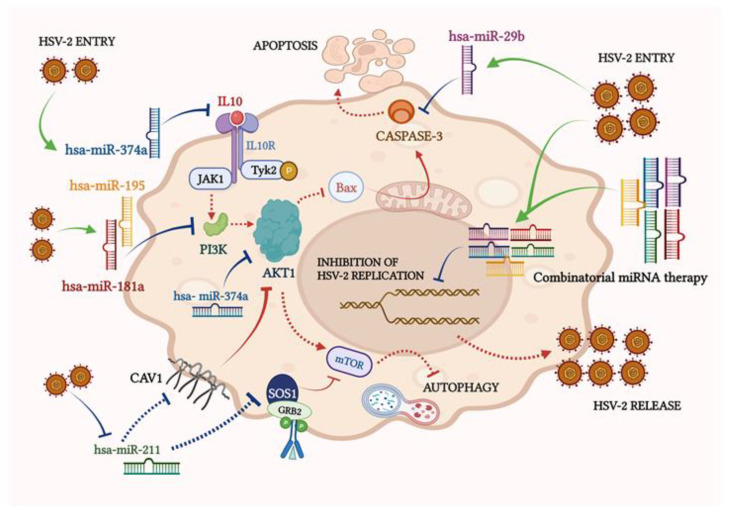
miRNAs in the HSV-2 infection of macrophages. HSV-2 infection of the macrophages elevates the expressions of hsa-miR-374a, -miR-29b, -miR-195 and miR-181a, and encourages the suppression of their respective target expressions. With the suppression of the targets of miR-374a, miR-195 and miR-181a, i.e., IL10, AKT1 and PI3K, AKT1 is ultimately suppressed, and is therefore, unable to inhibit autophagy. Suppression of AKT1 should have activated Bax-induced Caspase 3 elevation to trigger apoptosis, however, the elevated levels of miR-29b inhibit Caspase 3 in HSV-2 infection. Similarly, HSV-2 represses the expression of miR-211, which targets SOS1 and CAV1. These two targets are not suppressed by miR-211 and are able to inhibit mTOR and AKT1, respectively, leading to induction of autophagy. Thus, autophagy may be induced in HSV-2 infection of the macrophages in many ways. Further, a combinatorial miRNA exposure before HSV-2 infection is able to decrease the viral replication in the THP-1 macrophages and therefore, restrict the release of new virus particles. The arrows represented in green and blue indicate the findings shown in this manuscript, where green arrows represent upregulation or activation and blue arrows represent suppression or inhibition. The arrows in red represent the pathways already reported before this manuscript. The dotted arrows are indicative of the events that would have occurred but were interrupted due to HSV-2 infection. All-in-all, the miRNAs recognized to be modulated by HSV-2 infection can be ectopically expressed and may work synergistically to reduce HSV-2 infection in the macrophages.

The capability of miRNAs to target more than one gene expression can be harnessed to amend the targets in the pathway critically curbed by HSV-2 infection. The PI3K/AKT/mTOR pathway being one such pathway was targeted in our study. In this study, we have established that HSV-2 manipulates the intermediates of the PI3K/AKT/mTOR pathway through cellular miRNAs that not only have the potential to restrict HSV-2 pathogenesis but also contribute to the overall health of the host by alleviating other HSV-2 related pathologies and infections (Figure 8).

Overall, our study takes a leap forward in the research of host–HSV-2 interaction, providing possible mechanisms to be further investigated in HSV-2 therapeutics using the combinatorial effect of the nanoformulation of our identified miRNAs in in vivo models. On this note, our study concludes by outlining the implications of manipulating cellular miRNAs, opening an avenue for the development of such preventative and/or therapeutic agents that may be employed to function solitarily or synergistically to massively decrease the HSV-2 burden in the host.

## Data Availability

All data generated or analyzed during this study are included in this published article [and its Supplementary Information Files]. Raw datasets [Western blots and others] used and/or analyzed during the current study and the enlarged versions of selected main figures are available as the ‘Supplementary Data—Additional File’.

## Supplementary Materials

The following supporting information can be downloaded at: https://www.mdpi.com/article/10.3390/vaccines11091488/s1.

## Abbreviations

HSV: herpes simplex virus; miRNAs: micro-ribonucleic acids; IE: immediate early (genes); ICP: infected cell protein; WHO: World Health Organization; FDA: Food and Drug Administration; RISC: RNA-induced silencing complex; Hpi: hours post infection; NET: neutrophil extracellular traps; HPV: human papilloma virus; HIV: human immunodeficiency virus; PIK3R1: Phosphatidylinositol 3-kinase regulatory subunit 1; IL: Interleukin; CASP: Caspase; CAV: Caveolin; SOS: Son of Sevenless; sc-miR: scrambled miR; ANOVA: analysis of variance; PIP2: Phosphatidylinositol (3,4)-bisphosphate; PIP3: Phosphatidylinositol (3,4,5)-trisphosphate; AMPK: Adenosine-5′-monophosphate-activated protein kinase; HEPES: 4-(2-hydroxyethyl)-1-piperazineethanesulfonic acid; PMA: Phorbol 12-myristate 13-acetate; BBD: Box–Behnken Design; FBS: fetal bovine serum; PBS: phosphate buffered saline; NFDM: non-fat dried milk.
