# Supplementary material for: Combinatorial Effects of miRNAs in HSV-2 Infection of Macrophages: An In Silico and In Vitro Integration Approach
Source: Vaccines (Basel). 2023 Sep 14;11(9):1488. doi: 10.3390/vaccines11091488 (PMC10537408; doi:10.3390/vaccines11091488)
Supplement: Supplementary file 1 [file vaccines-11-01488-s001.zip › Supplementary Figures S1 - S7.pdf]

A

<table>
<tr><th>Inflammation</th><th>Regulation of Acute-Phase Response</th><th>Regulation of Cytokine Metabolism</th><th>Regulation of Cytokine Receptors</th><th>Regulation of Cytokine</th><th>Regulation of Humoral Immune Response</th></tr>
<tr>
<td rowspan="2">hsa-miR-106b-5p
hsa-miR-15b-5p
hsa-miR-16-5p
hsa-miR-17-5p
hsa-miR-20a-5p
hsa-miR-23b-3p
hsa-miR-98-5p
hsa-miR-181a-5p
hsa-miR-181b-5p
hsa-miR-181c-5p
hsa-miR-19b-3p
Autoimmunity
hsa-let-7f-5p
hsa-miR-16-5p
hsa-miR-17-5p
hsa-miR-20a-5p
hsa-miR-23a-3p
hsa-miR-23b-3p
hsa-miR-29c-3p
hsa-miR-181a-5p
hsa-miR-181b-5p
hsa-miR-301a-3p</td>
<td rowspan="2">hsa-let-7f-5p
hsa-let-7g-5p
hsa-miR-106b-5p
hsa-miR-125a-5p
hsa-miR-17-5p
hsa-miR-20a-5p
hsa-miR-20b-5p
hsa-miR-21-5p
hsa-miR-29b-3p
hsa-miR-29c-3p
hsa-miR-374a-5p
hsa-miR-454-3p
hsa-miR-98-5p
hsa-miR-144-3p
hsa-miR-101-3p
hsa-let-7a-5p
hsa-miR-130a-3p
hsa-miR-130b-3p
hsa-miR-202-3p
hsa-miR-301a-3p
hsa-miR-410-3p
hsa-miR-449a
hsa-miR-548d-3p
Innate & Adaptive Immunity
hsa-miR-17-5p
hsa-miR-20a-5p
hsa-miR-9-5p
hsa-miR-181a-5p
hsa-miR-19b-3p</td>
<td rowspan="2">hsa-let-7f-5p
hsa-let-7g-5p
hsa-miR-125a-5p
hsa-miR-15b-5p
hsa-miR-16-5p
hsa-miR-181a-5p
hsa-miR-195-5p
hsa-miR-23a-3p
hsa-miR-23b-3p
hsa-miR-30c-5p
hsa-miR-30d-5p
hsa-miR-374a-5p
hsa-miR-424-5p
hsa-miR-454-3p
hsa-miR-9-5p
hsa-miR-98-5p
hsa-let-7a-5p
hsa-miR-130a-3p
hsa-miR-130b-3p
hsa-miR-15a-5p
hsa-miR-181b-5p
hsa-miR-181c-5p
hsa-miR-19b-3p
hsa-miR-202-3p
hsa-miR-301a-3p
hsa-miR-302a-3p
hsa-miR-302c-3p
hsa-miR-410-3p
hsa-miR-449a
hsa-miR-543</td>
<td colspan="2">hsa-let-7f-5p; hsa-let-7g-5p
hsa-miR-106b-5p; hsa-miR-125a-5p
hsa-miR-15b-5p; hsa-miR-16-5p
hsa-miR-17-5p; hsa-miR-195-5p
hsa-miR-20a-5p; hsa-miR-20b-5p
hsa-miR-21-5p; hsa-miR-23a-3p
hsa-miR-23b-3p; hsa-miR-29b-3p
hsa-miR-29c-3p; hsa-miR-30c-5p
hsa-miR-30d-5p; hsa-miR-424-5p
hsa-miR-98-5p; hsa-miR-454-3p
hsa-miR-9-5p
hsa-let-7a-5p; hsa-miR-15a-5p
hsa-miR-19b-3p; hsa-miR-202-3p
hsa-miR-656-3p; hsa-miR-130a-3p
hsa-miR-130b-3p; hsa-miR-211-5p
hsa-miR-301a-3p; hsa-miR-548d-3p</td>
<td rowspan="2">hsa-let-7f-5p
hsa-let-7g-5p
hsa-miR-106b-5p
hsa-miR-125a-5p
hsa-miR-15b-5p
hsa-miR-16-5p
hsa-miR-17-5p
hsa-miR-195-5p
hsa-miR-20a-5p
hsa-miR-20b-5p
hsa-miR-21-5p
hsa-miR-23a-3p
hsa-miR-23b-3p
hsa-miR-29b-3p
hsa-miR-29c-3p
hsa-miR-30c-5p
hsa-miR-30d-5p
hsa-miR-424-5p
hsa-miR-98-5p
hsa-miR-181a-5p
hsa-miR-374a-5p
hsa-let-7a-5p
hsa-miR-15a-5p
hsa-miR-19b-3p
hsa-miR-202-3p
hsa-miR-656-3p
hsa-miR-181b-5p
hsa-miR-181c-5p
hsa-miR-302a-3p
hsa-miR-302c-3p
hsa-miR-410-3p
hsa-miR-543</td>
</tr>
<tr>
<td>hsa-miR-381-3p
hsa-miR-449a</td>
<td>hsa-miR-181a-5p
hsa-miR-374a-5p
hsa-miR-15a-5p
hsa-miR-181b-5p
hsa-miR-181c-5p
hsa-miR-302a-3p
hsa-miR-302c-3p
hsa-miR-410-3p
hsa-miR-543
hsa-miR-301b-3p
hsa-miR-144-3p
hsa-miR-101-3p</td>
</tr>
</table>

B

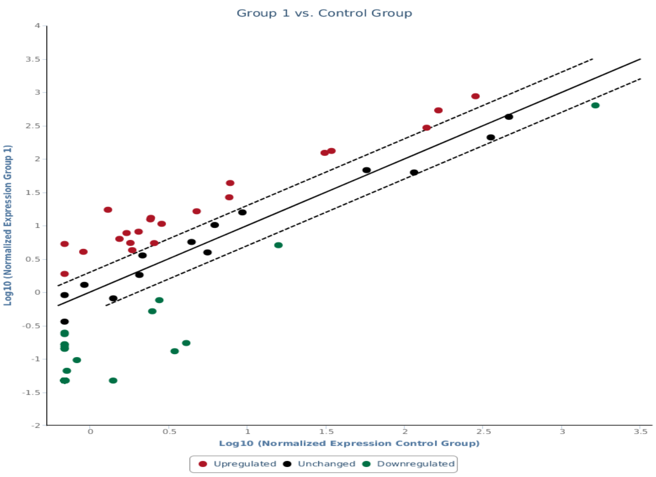

C

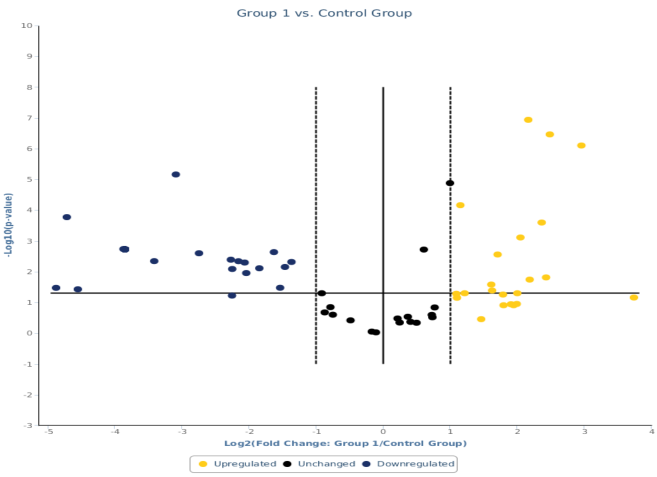

Figure S1

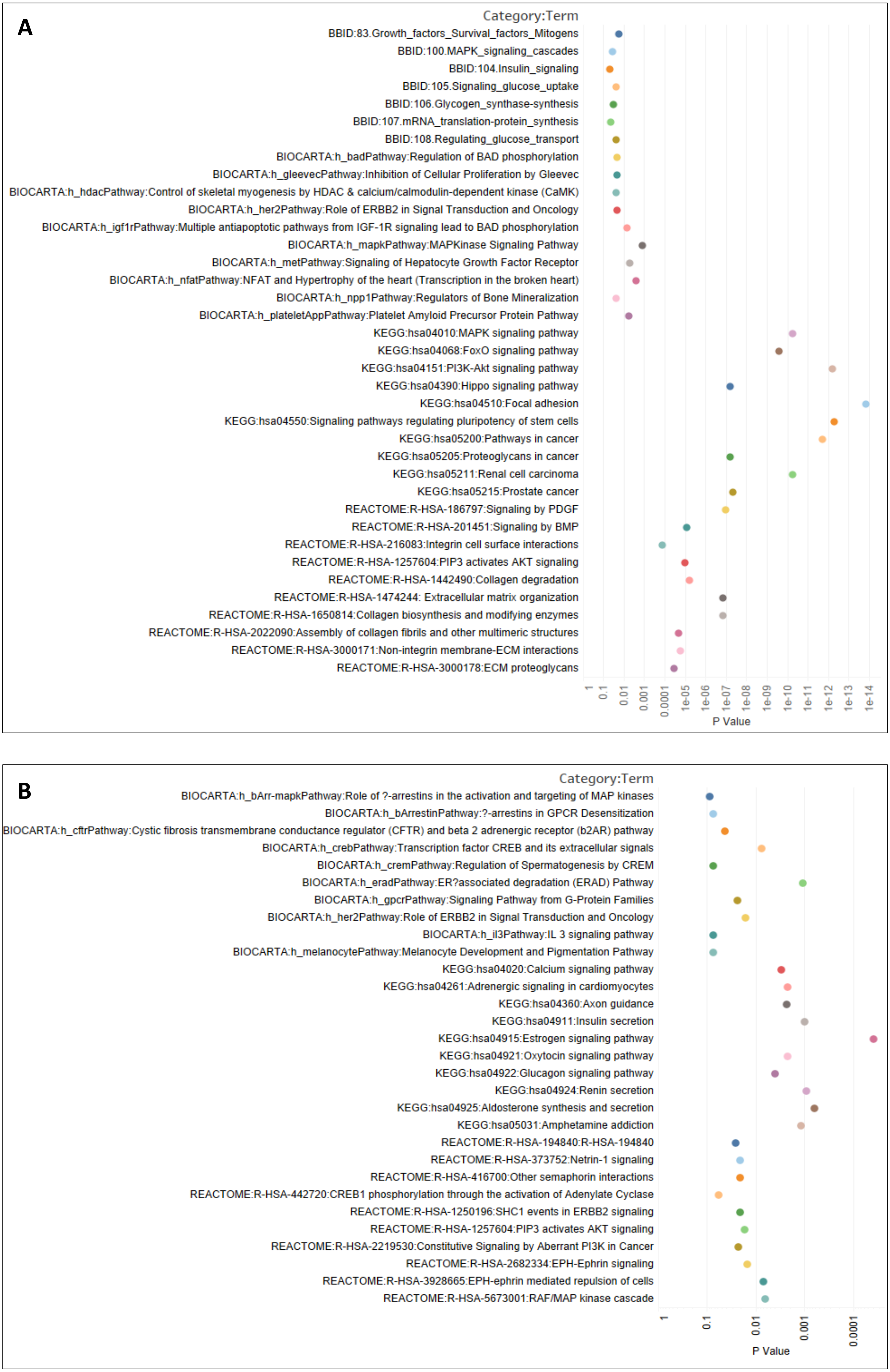
A
Category:Term
BBID:83.Growth_factors_Survival_factors_Mitogens
BBID:100.MAPK_signaling_cascades
BBID:104.Insulin_signaling
BBID:105.Signaling_glucose_uptake
BBID:106.Glycogen_synthase-synthesis
BBID:107.mRNA_translation-protein_synthesis
BBID:108.Regulating_glucose_transport
BIOCARTA:h_badPathway:Regulation of BAD phosphorylation
BIOCARTA:h_gleevecPathway:Inhibition of Cellular Proliferation by Gleevec
BIOCARTA:h_hdacPathway:Control of skeletal myogenesis by HDAC & calcium/calmodulin-dependent kinase (CaMK)
BIOCARTA:h_her2Pathway:Role of ERBB2 in Signal Transduction and Oncology
BIOCARTA:h_igf1rPathway:Multiple antiapoptotic pathways from IGF-1R signaling lead to BAD phosphorylation
BIOCARTA:h_mapkPathway:MAPKinase Signaling Pathway
BIOCARTA:h_metPathway:Signaling of Hepatocyte Growth Factor Receptor
BIOCARTA:h_nfatPathway:NFAT and Hypertrophy of the heart (Transcription in the broken heart)
BIOCARTA:h_npp1Pathway:Regulators of Bone Mineralization
BIOCARTA:h_plateletAppPathway:Platelet Amyloid Precursor Protein Pathway
KEGG:hsa04010:MAPK signaling pathway
KEGG:hsa04068:FoxO signaling pathway
KEGG:hsa04151:PI3K-Akt signaling pathway
KEGG:hsa04390:Hippo signaling pathway
KEGG:hsa04510:Focal adhesion
KEGG:hsa04550:Signaling pathways regulating pluripotency of stem cells
KEGG:hsa05200:Pathways in cancer
KEGG:hsa05205:Proteoglycans in cancer
KEGG:hsa05211:Renal cell carcinoma
KEGG:hsa05215:Prostate cancer
REACTOME:R-HSA-186797:Signaling by PDGF
REACTOME:R-HSA-201451:Signaling by BMP
REACTOME:R-HSA-216083:Integrin cell surface interactions
REACTOME:R-HSA-1257604:PIP3 activates AKT signaling
REACTOME:R-HSA-1442490:Collagen degradation
REACTOME:R-HSA-1474244: Extracellular matrix organization
REACTOME:R-HSA-1650814:Collagen biosynthesis and modifying enzymes
REACTOME:R-HSA-2022090:Assembly of collagen fibrils and other multimeric structures
REACTOME:R-HSA-3000171:Non-integrin membrane-ECM interactions
REACTOME:R-HSA-3000178:ECM proteoglycans
1
0.1
0.01
0.001
0.0001
1e-05
1e-06
1e-07
1e-08
1e-09
1e-10
1e-11
1e-12
1e-13
1e-14
P Value
B
Category:Term
BIOCARTA:h_bArr-mapkPathway:Role of ?-arrestins in the activation and targeting of MAP kinases
BIOCARTA:h_bArrestinPathway:?-arrestins in GPCR Desensitization
BIOCARTA:h_cftrPathway:Cystic fibrosis transmembrane conductance regulator (CFTR) and beta 2 adrenergic receptor (b2AR) pathway
BIOCARTA:h_crebPathway:Transcription factor CREB and its extracellular signals
BIOCARTA:h_cremPathway:Regulation of Spermatogenesis by CREM
BIOCARTA:h_eradPathway:ER?associated degradation (ERAD) Pathway
BIOCARTA:h_gpcrPathway:Signaling Pathway from G-Protein Families
BIOCARTA:h_her2Pathway:Role of ERBB2 in Signal Transduction and Oncology
BIOCARTA:h_il3Pathway:IL 3 signaling pathway
BIOCARTA:h_melanocytePathway:Melanocyte Development and Pigmentation Pathway
KEGG:hsa04020:Calcium signaling pathway
KEGG:hsa04261:Adrenergic signaling in cardiomyocytes
KEGG:hsa04360:Axon guidance
KEGG:hsa04911:Insulin secretion
KEGG:hsa04915:Estrogen signaling pathway
KEGG:hsa04921:Oxytocin signaling pathway
KEGG:hsa04922:Glucagon signaling pathway
KEGG:hsa04924:Renin secretion
KEGG:hsa04925:Aldosterone synthesis and secretion
KEGG:hsa05031:Amphetamine addiction
REACTOME:R-HSA-194840:R-HSA-194840
REACTOME:R-HSA-373752:Netrin-1 signaling
REACTOME:R-HSA-416700:Other semaphorin interactions
REACTOME:R-HSA-442720:CREB1 phosphorylation through the activation of Adenylate Cyclase
REACTOME:R-HSA-1250196:SHC1 events in ERBB2 signaling
REACTOME:R-HSA-1257604:PIP3 activates AKT signaling
REACTOME:R-HSA-2219530:Constitutive Signaling by Aberrant PI3K in Cancer
REACTOME:R-HSA-2682334:EPH-Ephrin signaling
REACTOME:R-HSA-3928665:EPH-ephrin mediated repulsion of cells
REACTOME:R-HSA-5673001:RAF/MAP kinase cascade
1
0.1
0.01
0.001
0.0001
P Value

Figure S2

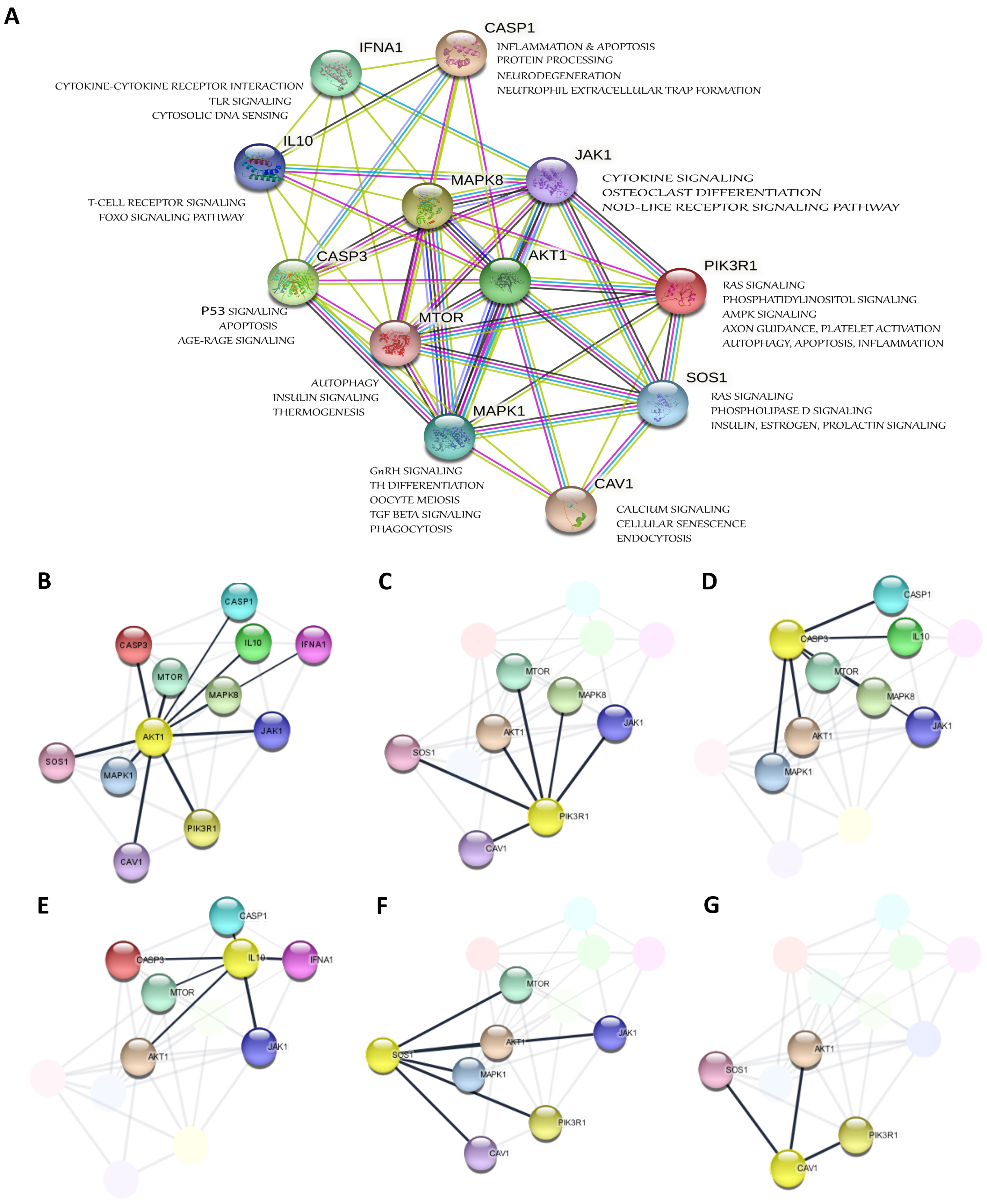
A
IFNA1
CASP1
INFLAMMATION & APOPTOSIS
PROTEIN PROCESSING
NEURODEGENERATION
NEUTROPHIL EXTRACELLULAR TRAP FORMATION
CYTOKINE-CYTOKINE RECEPTOR INTERACTION
TLR SIGNALING
CYTOSOLIC DNA SENSING
IL10
JAK1
CYTOKINE SIGNALING
OSTEOCLAST DIFFERENTIATION
NOD-LIKE RECEPTOR SIGNALING PATHWAY
MAPK8
T-CELL RECEPTOR SIGNALING
FOXO SIGNALING PATHWAY
CASP3
AKT1
PIK3R1
RAS SIGNALING
PHOSPHATIDYLINOSITOL SIGNALING
AMPK SIGNALING
AXON GUIDANCE, PLATELET ACTIVATION
AUTOPHAGY, APOPTOSIS, INFLAMMATION
P53 SIGNALING
APOPTOSIS
AGE-RAGE SIGNALING
MTOR
SOS1
RAS SIGNALING
PHOSPHOLIPASE D SIGNALING
INSULIN, ESTROGEN, PROLACTIN SIGNALING
AUTOPHAGY
INSULIN SIGNALING
THERMOGENESIS
MAPK1
GnRH SIGNALING
TH DIFFERENTIATION
OOCYTE MEIOSIS
TGF BETA SIGNALING
PHAGOCYTOSIS
CAV1
CALCIUM SIGNALING
CELLULAR SENESCENCE
ENDOCYTOSIS
B
CASP1
CASP3
IL10
IFNA1
MTOR
MAPK8
AKT1
JAK1
SOS1
MAPK1
PIK3R1
CAV1
C
MTOR
MAPK8
AKT1
JAK1
SOS1
PIK3R1
CAV1
D
CASP1
CASP3
IL10
MTOR
MAPK8
AKT1
JAK1
MAPK1
E
CASP1
CASP3
IL10
IFNA1
MTOR
AKT1
JAK1
F
MTOR
AKT1
JAK1
SOS1
MAPK1
PIK3R1
CAV1
G
AKT1
SOS1
PIK3R1
CAV1

Figure S3

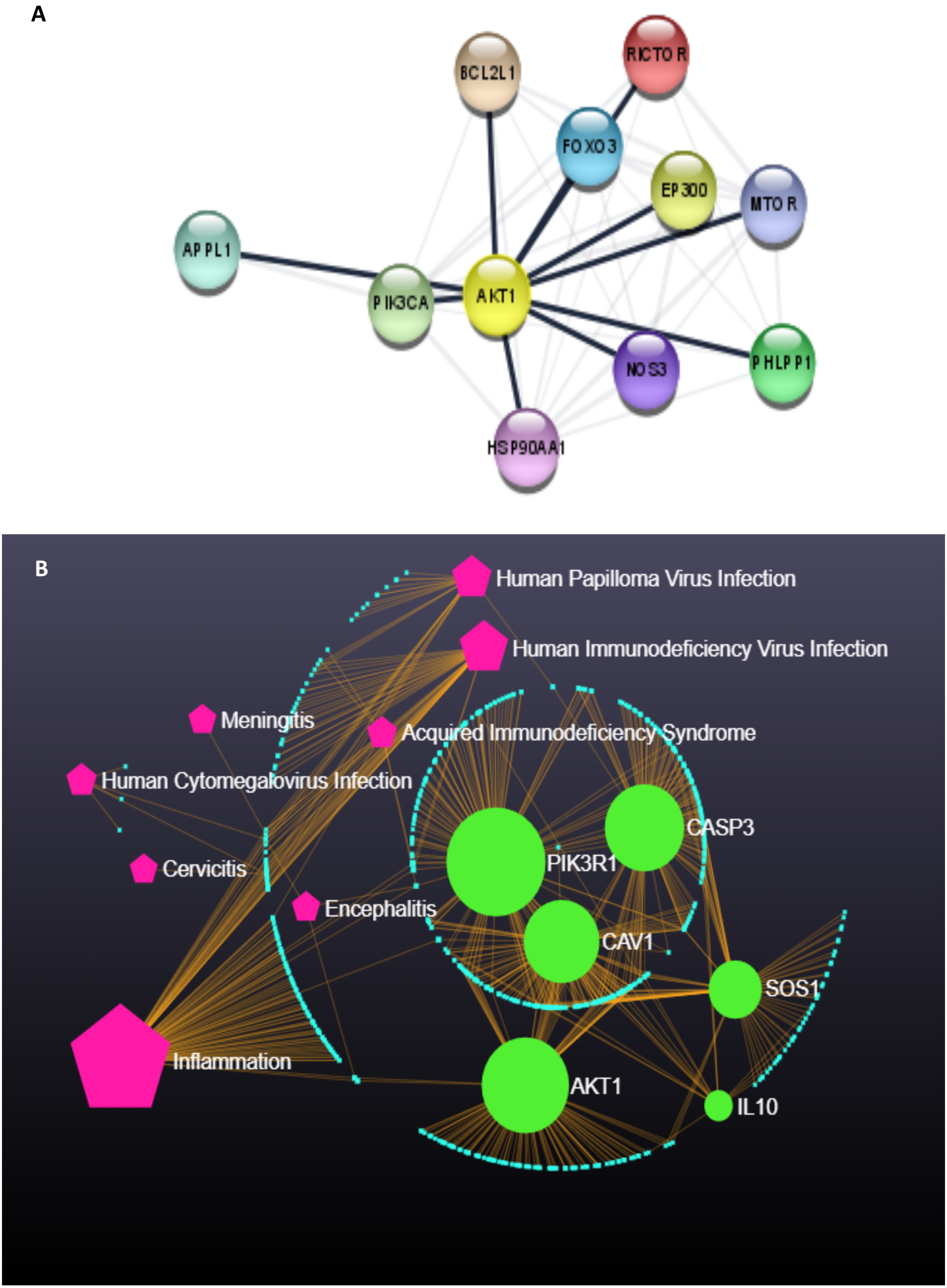
A
RICTOR
BCL2L1
FOXO3
EP300
MTOR
APPL1
PIK3CA
AKT1
NOS3
PHLPP1
HSP90AA1
B
Human Papilloma Virus Infection
Human Immunodeficiency Virus Infection
Meningitis
Acquired Immunodeficiency Syndrome
Human Cytomegalovirus Infection
CASP3
PIK3R1
Cervicitis
Encephalitis
CAV1
SOS1
Inflammation
AKT1
IL10

Figure S4

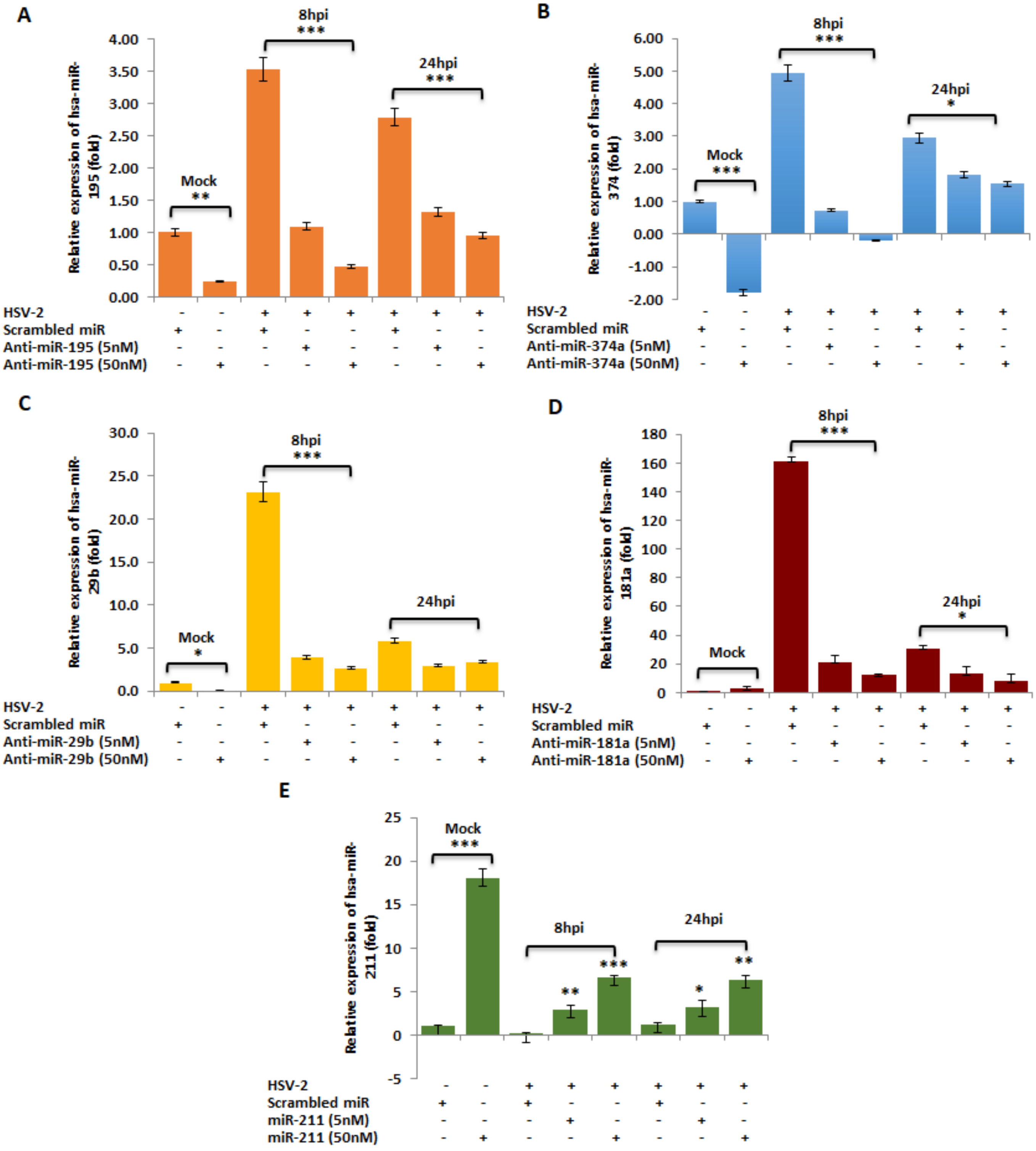
A
Relative expression of hsa-miR-195 (fold)
Mock
8hpi
24hpi
HSV-2
Scrambled miR
Anti-miR-195 (5nM)
Anti-miR-195 (50nM)
B
Relative expression of hsa-miR-374 (fold)
Mock
8hpi
24hpi
HSV-2
Scrambled miR
Anti-miR-374a (5nM)
Anti-miR-374a (50nM)
C
Relative expression of hsa-miR-29b (fold)
Mock
8hpi
24hpi
HSV-2
Scrambled miR
Anti-miR-29b (5nM)
Anti-miR-29b (50nM)
D
Relative expression of hsa-miR-181a (fold)
Mock
8hpi
24hpi
HSV-2
Scrambled miR
Anti-miR-181a (5nM)
Anti-miR-181a (50nM)
E
Relative expression of hsa-miR-211 (fold)
Mock
8hpi
24hpi
HSV-2
Scrambled miR
miR-211 (5nM)
miR-211 (50nM)

Figure S5

A

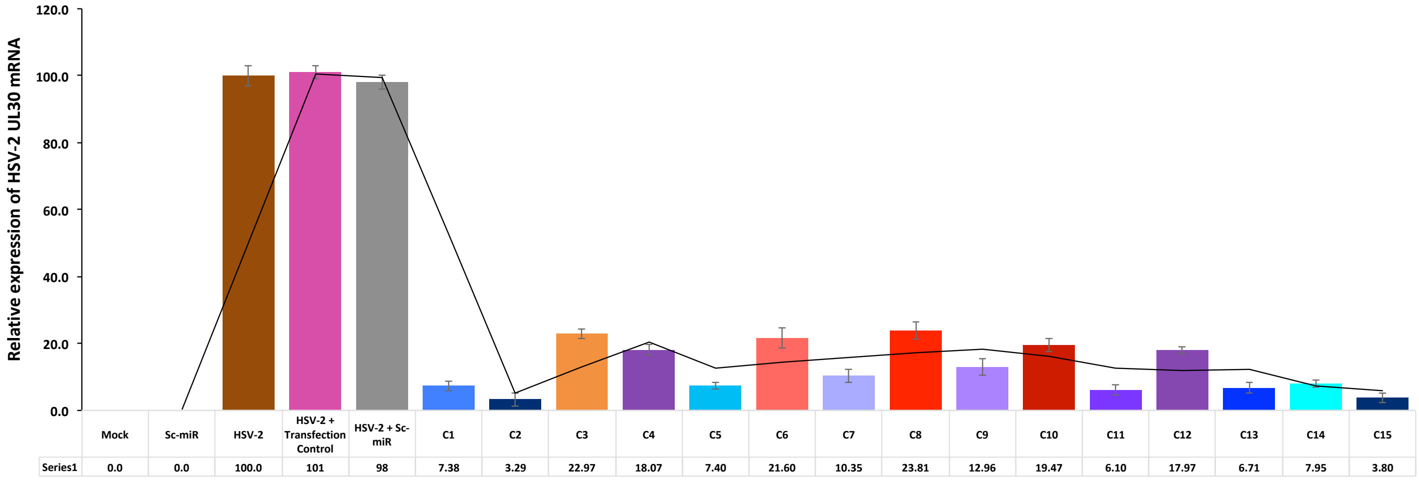

B

Combination Name	STD	RUN	Anti-mIR-195	Anti-mIR-374a	Anti-mIR-29b	Anti-mIR-181a	mimic-mIR-211	Total conc. In combination (nM)	Working Conc.	% Inhibition of HSV-2
**C1**	14	1	46.44	36.46	15.99	28.37	28.055	**155.315**	**50nM**	**92.61652181**
**C2**	7	4	32.44	36.46	15.99	41.37	28.055	**154.315**	**50nM**	**96.70510872**
**C3**	35	7	18.44	36.46	28.99	28.37	41.055	**153.315**	**50nM**	**77.02890145**
**C4**	26	10	46.44	36.46	28.99	15.37	28.055	**155.315**	**50nM**	**81.9314074**
**C5**	5	12	32.44	36.46	15.99	15.37	28.055	**128.315**	**50nM**	**92.60462644**
**C6**	10	14	32.44	49.46	28.99	28.37	15.055	**154.315**	**50nM**	**78.39562524**
**C7**	3	17	18.44	49.46	28.99	28.37	28.055	**153.315**	**50nM**	**89.65484778**
**C8**	11	20	32.44	23.46	28.99	28.37	41.055	**154.315**	**50nM**	**76.1929483**
**C9**	16	22	46.44	36.46	41.99	28.37	28.055	**181.315**	**50nM**	**87.03932045**
**C10**	6	25	32.44	36.46	41.99	15.37	28.055	**154.315**	**50nM**	**80.53365204**
**C11**	33	28	18.44	36.46	28.99	28.37	15.055	**127.315**	**50nM**	**93.90499636**
**C12**	8	30	32.44	36.46	41.99	41.37	28.055	**180.315**	**50nM**	**82.02954372**
**C13**	20	35	32.44	36.46	28.99	41.37	41.055	**180.315**	**50nM**	**93.28829546**
**C14**	21	40	32.44	23.46	15.99	28.37	28.055	**128.315**	**50nM**	**92.04539284**
**C15**	29	42	32.44	36.46	15.99	28.37	15.055	**128.315**	**50nM**	**96.19967089**

Figure S6

A

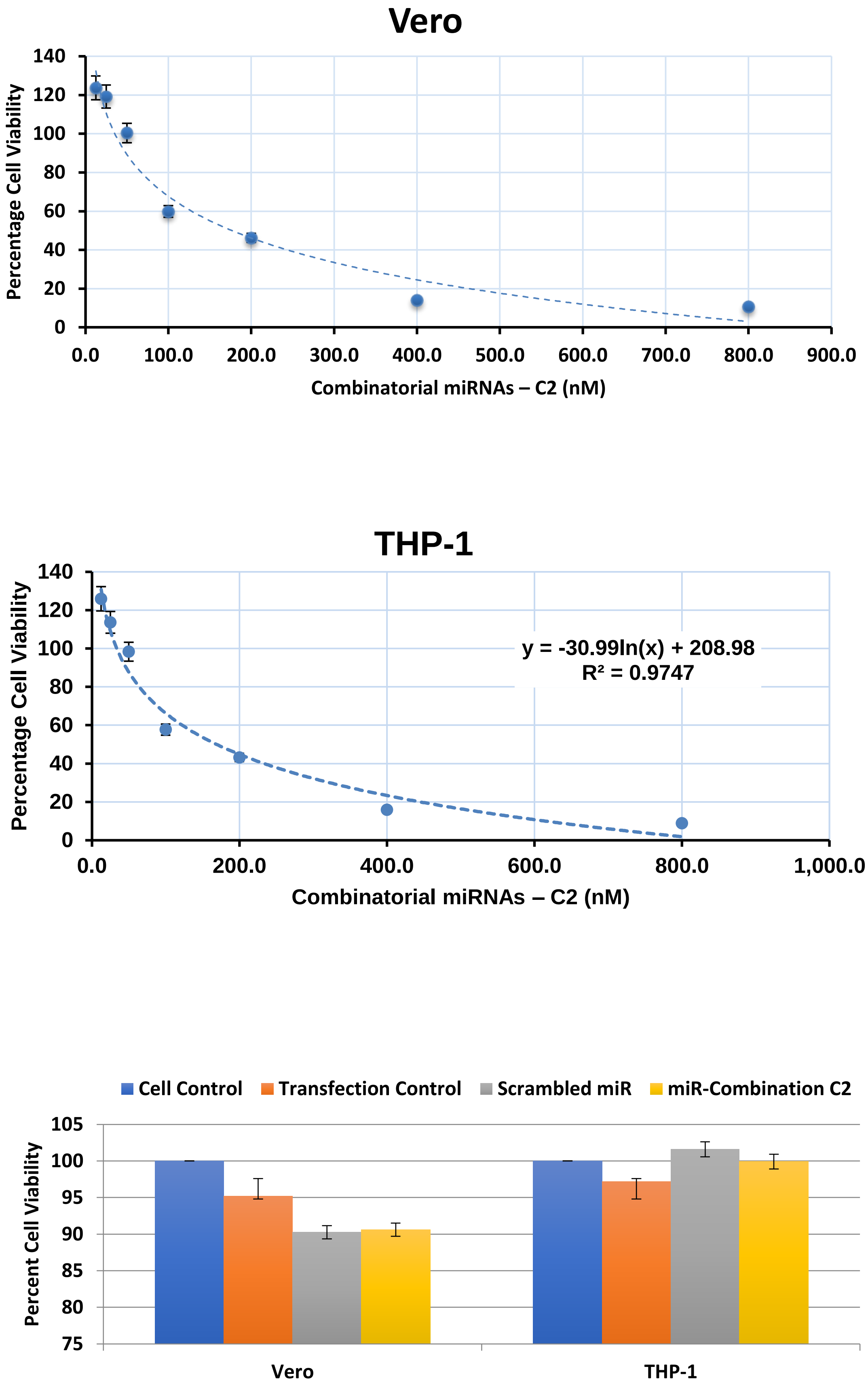
Vero
Percentage Cell Viability
140
120
100
80
60
40
20
0
0.0
100.0
200.0
300.0
400.0
500.0
600.0
700.0
800.0
900.0
Combinatorial miRNAs – C2 (nM)
B
THP-1
y = -30.99ln(x) + 208.98
R² = 0.9747
1,000.0
C
Cell Control
Transfection Control
Scrambled miR
miR-Combination C2
Percent Cell Viability
105
95
90
85
80
75
THP-1

Figure S7

Supplementary Figure Legends: S1-S7

Figure S1: (A) The cellular processes or events associated with the upregulated and the downregulated miRNAs. The upregulated miRNAs in the HSV-2 infected macrophage samples are represented in red colour while downregulated ones are represented in green. Different representations -(B) Scatter plot (C) Volcano plot- of 43 dysregulated miRNAs in the human inflammatory response pathway-focused miRNA microarray assay using Control Vs. HSV-2 infected samples. Red and yellow indicate the upregulated while green and blue represent the downregulated miRNAs in the scatter and volcano plot, respectively. 43 miRNAs were observed to be differentially expressed between the Control and the HSV-2 infected samples at the $P^* < 0.05$ significance level.

Figure S2: Top ten over-represented pathways associated with (A) targets of the overexpressed miRNAs and (B) targets of the under-expressed miRNA, as per functional enrichment analysis performed against different pathway databases using DAVID.

Figure S3: Gene neighborhood of the selected miRNA targets. (A) STRING Network analysis to reveal the focal point of the target interaction network. The colored nodes represent the miRNA targets and their interactors. Structures within the nodes indicate known 3D structure of their proteins. Different colored edges denote known or predicted interactions from curated databases or are experimentally determined, with fusions, neighborhood, co-occurrence, protein homology or co-expression between them (B-G) First neighbors of each of the miRNA targets. All the network analysis was done using the StringApp in Cytoscape 3.8.2.

Figure S4: (A) String network analysis of the known interactions of AKT1 with other target proteins. (B) miRNet visual analytics platform to represent the interaction network of the targets with other miRNAs and the diseases that may become associated with HSV-2 infection. The green, circular nodes represent the miRNA targets, the blue small, circular nodes represent the miRNAs that are already known to target them and the pink, pentagonal nodes represent the diseases that are linked with HSV-2 infection. The edges in orange represent the interactions between the miRNAs, their targets and the HSV-2 associated diseases.

Figure S5: Transfection efficiencies of the mimic or inhibitors of the miRNAs. (A-D) The fold-change in relative expression of the miRNAs in samples transfected with inhibitors of miR-195, miR-374a, miR-29b or miR-181a at 5nM or 50nM transfected concentrations. (E) The fold-change in relative expression of the miRNAs in samples transfected with the mimic of miR-211 at 5nM or 50nM transfected concentrations.

Figure S6: Representation of *in vitro* validation of the selected 15 miRNA combinations based on the degree of HSV-2 inhibition. Out of the 15 combinations tested for their relative inhibitory effect on the HSV-2 UL30 gene transcription, the highest relative inhibitory effect was observed in the C2 combination (~97%), followed by C15 (~96%), with the lowest inhibition obtained by the combination C8 (~76%), when compared with the HSV-2 infected but untransfected samples or samples without treatment. No change in the UL30 expression was observed upon transfection of transfection control or Scrambled miR followed by HSV-2 infection when compared to the untransfected HSV-2 infected samples.

Figure S7: Cell viability assay by MTT. The effect of different concentrations of the miRNA combination C2 on (A) Vero epithelial cells and (B) differentiated THP-1 cells. (C) The mean CC_{50} value of miRNA combination C2 (Yellow) as determined from three independent assays, in comparison to the different controls used in the assay, i.e. Untransfected cell control (Blue), Transfection control-transfected (Orange) and Scrambled miR-transfected (Grey).
