# Supplementary material for: Combinatorial Effects of miRNAs in HSV-2 Infection of Macrophages: An In Silico and In Vitro Integration Approach
Source: Vaccines (Basel). 2023 Sep 14;11(9):1488. doi: 10.3390/vaccines11091488 (PMC10537408; doi:10.3390/vaccines11091488)
Supplement: Supplementary file 1 [file vaccines-11-01488-s001.zip › Supplementary Tables S1 - S10.pdf]

Supplementary Information
Additional File 1: Tables S1-S10

Supplementary Table S1: Primer sequences used in the SYBR Green based qPCR assays involving amplification of HSV-2 viral genes

Sl.no.	*Gene Type*	*Primer name*	*Primer sequence*
**1.**	**Ubiquitin E3 ligase ICP0**	α-HSV-2-**RL2-F**	5' -GTTCAGCACCATCCCGATA- 3'
**2.**		α-HSV-2-**RL2-R**	5' -GGTTGCCCGTCCAGATAAA- 3'
**3.**	**DNA polymerase catalytic subunit**	α-HSV-2-**UL30-F**	5'- CGCTCAACACGGACTATTACTT- 3'
**4.**		α-HSV-2-**UL30-R**	5' -CTCGGTGATCTTGGCGTTATT- 3'
**5.**	**Glycoprotein C**	α-HSV-2-**UL44-F**	5' -ATGCCGGTTTCCCAACTC- 3'
**6.**		α-HSV-2-**UL44-R**	5' -CGTTTACCATCACCTCCTCTAAG- 3'
**7.**	**Internal Control**	**GAPDH-F**	5' -CAAGAGCACAAGAGGAAGAGAG- 3'
**8.**		**GAPDH-R**	5'- CTACATGGCAACTGTGAGGAG- 3'

Supplementary Table S2: Primer sequences used for cloning the 3' UTR of the target genes for target validation using Luciferase Assay. The red and the green coloured regions represent the restriction sites for the enzymes MLU1and HINDIII, respectively.

Sl.no.	*miRNA : Gene Target*	*Primer name*	*Primer sequence*
**1.**	miR-29b :	3'UTR-**CASPASE-3_F**	5'-GAGAACGCGTGAGCAAACTAACTTGAC -3'
**2.**	**Caspase-3**	3'UTR-**CASPASE-3_R**	5'-GTATATGAAGCTTAACTGATATGCATAGGGACAAATC -3'
**3.**	miR-181a :	3'UTR-**PIK3R1_F**	5'-CTGATACGCGTCAAAGCTGCTTTATTC -3'
**4.**	**PIK3R1**	3'UTR-**PIK3R1_R**	5'- AGTAGAAGAAGCTTCTTCACTTTGCAGTTATTC -3'
**5.**	miR-195 :	3'UTR-**PIK3R1_F**	5'- GAAACGCGTCACCATGAGATAGCATTA -3'
**6.**	**PIK3R1**	3'UTR-**PIK3R1_R**	5'- CATTGAAAAGCTTGTAGCTTTAAAAAATACACAGG -3'
**7.**	miR-374a :	3'UTR-**AKT1_F**	5'- TTTTCGACGCGTAACCTTTCCG -3'
**8.**	**AKT1**	3'UTR-**AKT1_R**	5'- AGCAAGCTTTATTGAAGAATTTGGAGGGAAG -3'
**9.**	miR-374a :	3'UTR-**IL10_F**	5'- TTACTACGCGTCTCTGTGAACGATTTAGAAAG -3'
**10.**	**IL10**	3'UTR-**IL10_R**	5'- GTCAAGCTTGGAATGGAAGCTTCTGTT -3'
**11.**	miR-211 :	3'UTR-**SOS1_F**	5'- AACGACGCGTCTTCAAGATGCAGTATAA -3'
**12.**	**SOS1**	3'UTR-**SOS1_R**	5'- ACAAAAGCTTTCATGTGGGCCTG -3'
**13.**	miR-211 :	3'UTR-**CAV1_F**	5'- TTGCACGCGTCTGTTATGCTGTGA -3'
**14.**	**CAV1**	3'UTR-**CAV1_R**	5'- AGGAAGCTTTCTGTACGACTCAGG -3'

Supplementary Table S3: Variables identified based on the initial *in vitro* viral inhibition used in Box–Behnken Design

**Factor**	**Name**	**Minimum**	**Maximum**	**Coded Low**	**Coded High**	**Mean**	**Std. Dev.**
**A**	anti-miR-195	18.44	46.44	-1 ↔ 18.44	+1 ↔ 46.44	32.44	8.64
**B**	anti-miR-374a	23.46	49.46	-1 ↔ 23.46	+1 ↔ 49.46	36.46	8.02
**C**	anti-miR-29b	15.99	41.99	-1 ↔ 15.99	+1 ↔ 41.99	28.99	8.02
**D**	anti-miR-181a	15.37	41.37	-1 ↔ 15.37	+1 ↔ 41.37	28.37	8.02
**E**	miR-211	15.05	41.05	-1↔ 15.05	+1 ↔ 41.05	28.05	8.02

Supplementary Table S4: *miRNA targets* common to the 4 upregulated miRNAs, or any 3 of the upregulated miRNAs

**Common Targets (All 4 miRNAs)**	**Common Targets (miR-374, miR-29b, miR-181a)**	**Common Targets (miR-195, miR-29b, miR-181a)**	**Common Targets (miR-195, miR-374, miR-181a)**	**Common Targets (miR-195, miR-374, miR-29b)**
PTPN4	N4BP2	DCLK1	TNRC6B	ZBTB46
PLAG1	CAMSAP2	AKT3	KIF1B	CPEB3
ZBTB34	ABHD18	DDX3X	IPO7	AK4
USP42	DLG2	RPS6KA3	ARMH4	USP31
RNF217	MED12L	PLXNC1	RFX3	DENND1B
ACVR2B	B3GNT5	ACVR2A	LATS1	BCL11B
ADAMTS6	NEGR1	KCNK10	CBX4	LRP6
RORA	SRSF10	CLOCK	STXBP5	ZBTB20
NOTCH2	CAND1	TLL1	ZNRF2	DENND4A
ZNF704	CRISPLD1	ELMSAN1	ACTR2	MIB1
PRKG1	RALA	JARID2	G2E3	PRDM11
NTRK2	YTHDF3	ILDR2	POU2F1	PHACTR2
KCNC2	PGAP1	MED26	PPM1A	SCAI
ZDHHC21	ADAMTS19	CAPRIN1	RPS6KA6	NEDD9
TMEM33	ZBTB41	PRRC2C	SPTLC1	RIC1
NEXMIF	VPS36	PIK3R1	ETNK1	C16orf72
BRWD1	TET2	DDX3Y	TCAIM	AFF4
	REST	RAB30	TSC22D2	IPO9
	DCAF12L1	SLC2A14	CHIC1	LDLRAD4
	RMND5A	SLC2A3	FAM135A	ZER1
	OSBPL3	OTUD4	ACSL4	UBE2K
	MEX3B	KPNA1	MBNL2	TFEC
	PLPPR4	PDIK1L	LCOR	CDK6
		CCNT2	YOD1	CLCN5
		E2F7	CRIM1	
		SPRY4	ADAMTS5	
		CCNJ	WSB1	
		ERLIN2	GABRA1	
		PCDHA8	SLITRK1	
		PCDHAC1	PLPP3	
		PCDHAC2	ZBTB43	
		PCDHA13	DCUN1D1	
		PCDHA10	PHIP	
		PCDHA5	PPP1R2	
		PCDHA6	ELAVL4	
		PCDHA11	SLC4A8	
		PCDHA3	TBC1D9	
		PCDHA4	RAB3IP	
		PCDHA1	PIK3C2A	
		PCDHA7	AK9	
		PCDHA2	ZNF699	
		PCDHA12	ONECUT2	
		KLF7	PSMF1	
		BACH2		
		PCDHA9		
		ARMC8		
		PTEN		

Supplementary Table S5: Top 50 miRNA targets sorted on the basis of degree value in the interaction network. The targets highlighted (in bold) were selected based on the pathway analysis and functional annotation and validated in this study

Top Over-expressed miRNA targets	*Degree*	*Top under-expressed miRNA targets*	*Degree*
**AKT1**	**522**	CCND1	70
VEGFA	310	ESR1	70
MAPK1	309	CREB1	68
EP300	291	**CAV1**	**59**
PTEN	284	PTPRC	58
CDC42	281	ERBB2	55
STAT3	279	BDNF	50
TNF	277	CDK1	47
CCND1	259	SIRT1	42
ESR1	257	HTT	42
KRAS	253	NTRK2	42
MAPK8	249	JAK2	41
APP	240	CUL3	38
CDK1	235	SHC1	37
**CASP3**	**225**	CDH2	37
HSPA8	224	PRKACB	36
BDNF	216	**SOS1**	**36**
SIRT1	214	RUNX2	36
IGF1	212	ITPR1	35
AR	207	PDGFRB	34
FGF2	204	PPARGC1A	32
**PIK3R1**	**200**	GNAQ	32
CREB1	199	EZR	32
FOS	198	HGF	32
HSPA4	191	KITLG	31
RAC1	188	WNT2	31
MDM2	185	LAMP1	31
BTRC	183	SP1	30
NRAS	180	CACNA1C	30
PPP2R1A	177	PPP1CC	30
GRB2	174	CDK6	30
ATM	173	ERBB3	30
SMAD2	169	PIK3CB	29
FYN	168	GRM1	29
CDC5L	167	GABRG2	29
CUL3	167	POLR2F	29
CYCS	166	FBXW7	28
**IL10**	**166**	NR3C1	28
FBXW7	165	FLT1	27
UBE2D1	165	SRSF1	27
GSK3B	162	NEUROD1	27
IL2	158	CAMK2D	27
SNAP25	157	AP2A2	26
HGF	152	MCL1	26
ELOC	148	DLG3	26
PRKN	148	DNM2	26
IGF1R	145	GNAS	25
MAP2K1	145	MBNL1	25
VHL	145	ZEB1	25
CDC27	144	NES	25

Supplementary Table S6: The minimum free energy values of the miRNA-mRNA duplex predicted using the miRmap web interface

miRNA	Gene	ΔG duplex	ΔG binding
hsa-miR-181a-5p	PIK3R1	-12.1	-10.8805
hsa-miR-195-5p	PIK3R1	-13.1	-14.0455
hsa-miR-211-5p	CAV1	-10.7	-11.8219
hsa-miR-211-5p	SOS1	-18.8	-18.4258
hsa-miR-29b-3p	CASP3	-11.4	-12.3499
hsa-miR-374a-5p	AKT1	-4	-4.08062
hsa-miR-374a-5p	IL10	-3.6	-4.11916

Supplementary Table S7: Observed response in Box-Behnken analyses for percent HSV-2 inhibition

Std.	Run	Space Type	Factor 1	Factor 2	Factor 3	Factor 4	Factor 5	Response 1
			**A: anti-miR-195**(nM)	**B: anti-miR-374a**(nM)	**C: anti-miR-29b**(nM)	**D: anti-miR-181a**(nM)	**E: mimic-miR-211**(nM)	**% Inhibition of HSV-2 Infection**
**9**	1	IBFact	32.44	23.46	28.99	28.37	15.055	90.22922932
**27**	2	IBFact	18.44	36.46	28.99	41.37	28.055	89.41142473
**41**	3	Center	32.44	36.46	28.99	28.37	28.055	86.61599763
**30**	4	IBFact	32.44	36.46	41.99	28.37	15.055	84.55859281
**26**	5	IBFact	46.44	36.46	28.99	15.37	28.055	81.9314074
**37**	6	IBFact	32.44	23.46	28.99	15.37	28.055	83.3080115
**34**	7	IBFact	46.44	36.46	28.99	28.37	15.055	90.74178837
**28**	8	IBFact	46.44	36.46	28.99	41.37	28.055	90.84692146
**5**	9	IBFact	32.44	36.46	15.99	15.37	28.055	92.60462644
**13**	10	IBFact	18.44	36.46	15.99	28.37	28.055	91.36369646
**31**	11	IBFact	32.44	36.46	15.99	28.37	41.055	88.67340246
**4**	12	IBFact	46.44	49.46	28.99	28.37	28.055	87.12855668
**16**	13	IBFact	46.44	36.46	41.99	28.37	28.055	87.03932045
**2**	14	IBFact	46.44	23.46	28.99	28.37	28.055	87.53893532
**12**	15	IBFact	32.44	49.46	28.99	28.37	41.055	83.00276595
**42**	16	Center	32.44	36.46	28.99	28.37	28.055	86.61599763
**39**	17	IBFact	32.44	23.46	28.99	41.37	28.055	90.33436242
**33**	18	IBFact	18.44	36.46	28.99	28.37	15.055	93.90499636
**15**	19	IBFact	18.44	36.46	41.99	28.37	28.055	80.43280208
**25**	20	IBFact	18.44	36.46	28.99	15.37	28.055	82.38507381
**8**	21	IBFact	32.44	36.46	41.99	41.37	28.055	82.02954372
**36**	22	IBFact	46.44	36.46	28.99	28.37	41.055	83.92570363
**43**	23	Center	32.44	36.46	28.99	28.37	28.055	86.61599763
**23**	24	IBFact	32.44	23.46	41.99	28.37	28.055	81.35573977
**3**	25	IBFact	18.44	49.46	28.99	28.37	28.055	89.65484778
**17**	26	IBFact	32.44	36.46	28.99	15.37	15.055	86.51086454
**19**	27	IBFact	32.44	36.46	28.99	15.37	41.055	79.69477981
**35**	28	IBFact	18.44	36.46	28.99	28.37	41.055	77.02890145
**29**	29	IBFact	32.44	36.46	15.99	28.37	15.055	96.19967089
**18**	30	IBFact	32.44	36.46	28.99	41.37	15.055	93.53721546
**11**	31	IBFact	32.44	23.46	28.99	28.37	41.055	76.1929483
**10**	32	IBFact	32.44	49.46	28.99	28.37	15.055	78.39562524
**32**	33	IBFact	32.44	36.46	41.99	28.37	41.055	77.74250808
**14**	34	IBFact	46.44	36.46	15.99	28.37	28.055	92.61652181
**20**	35	IBFact	32.44	36.46	28.99	41.37	41.055	93.28829546
**1**	36	IBFact	18.44	23.46	28.99	28.37	28.055	86.10343859
**38**	37	IBFact	32.44	49.46	28.99	15.37	28.055	82.89763285
**6**	38	IBFact	32.44	36.46	41.99	15.37	28.055	80.53365204
**24**	39	IBFact	32.44	49.46	41.99	28.37	28.055	80.94536112
**7**	40	IBFact	32.44	36.46	15.99	41.37	28.055	96.70510872
**22**	41	IBFact	32.44	49.46	15.99	28.37	28.055	91.8762555
**40**	42	IBFact	32.44	49.46	28.99	41.37	28.055	89.92398377
**21**	43	IBFact	32.44	23.46	15.99	28.37	28.055	92.04539284

Supplementary Table S8: Fit summary obtained from Box-Behnken Design run on the suggested models

Source	Sequential p-value	Adjusted R²	Predicted R²	Remark
**Linear**	< 0.0001	0.6703	0.5997	Suggested
**2FI**	0.1364	0.7217	0.5060	
**Quadratic**	0.2680	0.7403	0.4558	
**Cubic**	0.6961	0.6878	-2.3303	Aliased

Supplementary Table S9: ANNOVA test on the linear model and resultant parameters

Source	Sum of Squares	df	Mean Square	F-value	p-value	Remark
**Model**	870.17	5	174.03	18.08	< 0.0001	Significant
A-anti-miR-195	8.24	1	8.24	0.8563	0.3608	
B-anti-miR-374a	0.6736	1	0.6736	0.0700	0.7928	
C- anti-miR-29b	477.94	1	477.94	49.65	< 0.0001	
D- anti-miR-181a	197.48	1	197.48	20.51	< 0.0001	
E-miR-211	185.84	1	185.84	19.30	< 0.0001	
**Residual**	356.18	37	9.63			
Lack of Fit	356.18	35	10.18			
Pure Error	0.0000	2	0.0000			
**Cor Total**	1226.34	42				

Supplementary Table S10: Composition of optimized formulations of miRNAs with the predicted and experimental values of responses

Optimized composition	Response variable	Experimental value	Predicted Value
anti-miR-195: **32.440**<br>anti-miR-374a: **36.460**<br>anti-miR-29b: **15.990**<br>anti-miR-181a: **41.370**<br>miR-211: **28.055**	R1- % Inhibition	**96.7051**	**95.5946**
